# Distinct Mechanisms for Induction and Tolerance Regulate the Immediate Early Genes Encoding Interleukin 1β and Tumor Necrosis Factor α

**DOI:** 10.1371/journal.pone.0070622

**Published:** 2013-08-01

**Authors:** Juraj Adamik, Kent Z. Q. Wang, Sebnem Unlu, An-Jey A. Su, Gillian M. Tannahill, Deborah L. Galson, Luke A. O’Neill, Philip E. Auron

**Affiliations:** 1 Department of Biological Sciences, Duquesne University, Pittsburgh, Pennsylvania, United States of America; 2 Department of Microbiology and Molecular Genetics, University of Pittsburgh School of Medicine, Pittsburgh, Pennsylvania, United States of America; 3 School of Biochemistry and Immunology, Trinity College Dublin, Dublin, Ireland; 4 Division of Hematology/Oncology, Department of Medicine, University of Pittsburgh Cancer Institute, University of Pittsburgh School of Medicine, Pittsburgh, Pennsylvania, United States of America; Albany Medical College, United States of America

## Abstract

Interleukin-1β and Tumor Necrosis Factor α play related, but distinct, roles in immunity and disease. Our study revealed major mechanistic distinctions in the Toll-like receptor (TLR) signaling-dependent induction for the rapidly expressed genes (*IL1B* and *TNF*) coding for these two cytokines. Prior to induction, *TNF* exhibited pre-bound TATA Binding Protein (TBP) and paused RNA Polymerase II (Pol II), hallmarks of poised immediate-early (IE) genes. In contrast, unstimulated *IL1B* displayed very low levels of both TBP and paused Pol II, requiring the lineage-specific Spi-1/PU.1 (Spi1) transcription factor as an anchor for induction-dependent interaction with two TLR-activated transcription factors, C/EBPβ and NF-κB. Activation and DNA binding of these two pre-expressed factors resulted in *de novo* recruitment of TBP and Pol II to *IL1B* in concert with a permissive state for elongation mediated by the recruitment of elongation factor P-TEFb. This Spi1-dependent mechanism for *IL1B* transcription, which is unique for a rapidly-induced/poised IE gene, was more dependent upon P-TEFb than was the case for the *TNF* gene. Furthermore, the dependence on phosphoinositide 3-kinase for P-TEFb recruitment to *IL1B* paralleled a greater sensitivity to the metabolic state of the cell and a lower sensitivity to the phenomenon of endotoxin tolerance than was evident for *TNF*. Such differences in induction mechanisms argue against the prevailing paradigm that all IE genes possess paused Pol II and may further delineate the specific roles played by each of these rapidly expressed immune modulators.

## Introduction

Genome-wide approaches provide important insight into general processes of gene regulation. However, such global studies may result in a less detailed examination of mechanisms affecting a small number of critical genes. For example, immediate-early (IE) rapidly-induced, genes have been suggested to depend upon paused RNA polymerase II (Pol II) arrested at a site approximately 50 bp downstream of the transcription start by engagement with specific arresting factors. This constitutively paused state is thought to poise the gene for IE elongation following an appropriate signal [1]. A number of innate immunity genes respond to specific external stimuli by rapid IE-like induction, requiring kinetic evaluation of specific cell types treated with specific conditions that have not been widely explored with regard to the mechanisms of transcription regulation. In addition, many of these genes are subject to transient expression resulting from epigenetic mechanisms which rapidly shut-down transcription and render the gene refractory to re-induction from a repeat of the same [2], but not of a distinct [3] stimulus. In an attempt to better understand the regulation of such induction, we have focused on human interleukin-1β (*IL1B*) and tumor necrosis factor α (*TNF*), two innate immunity genes which exhibit cell-type restriction, transient IE induction, and conflicting reports of refractory re-induction, also referred to as stimulant tolerance [2], [3], [4]. We employed a combined approach using: cell lines and primary cells; reporter transient transfection; chromatin immunoprecipitation; evaluation of transcript integrity; ectopic expression in a non-competent cell type; and comparison to mouse orthologs in order to determine that an array of mechanisms interplay to distinctly regulate these genes. Our kinetic approach provides novel insight into the changes associated with Pol II recruitment, pausing and chromatin dynamics along these genes. While transient induction of *TNF* was associated with the expected IE mechanism, involving release of the NELF elongation inhibitory factor from paused Pol II, activation of *IL1B* depended on *de novo* recruitment of both TATA binding protein (TBP) and RNA Polymerase II (Pol II) and likely contributed to a slight induction delay. TBP has previously been reported to directly interact with the monocyte lineage transcription factor Spi-1/PU.1 (Spi1) [5], consistent with our observation that it appeared to be recruited to *IL1B* by this factor. We also show that ectopic expression of Spi1, along with the Toll-like receptor (TLR) surrogate TRAF6 in a cell line incompetent for *IL1B* transcription, primed the endogenous genome for *IL1B* induction by remodeling promoter nucleosomes and generated an expanded nucleosome depleted region (NDR) that likely supports the recruitment of TBP and Pol II in a manner reminiscent of that observed in monocytes. In contrast to *TNF*, whose induction is primarily dependent upon NF-κB [6], *IL1B* is co-dependent on both C/EBPβ [7], [8], [9] and NF-κB [10], transcription factors simultaneously activated by lipopolysaccharide/Toll-like receptor 4 (LPS/TLR4) signaling [11], [12] In support of previous transient transfection and *in vitro* interaction studies arguing for a long range interaction between a far-upstream bound C/EBPβ and promoter bound Spi1 [13], [14], we observed a corresponding signal-dependent chromatin loop for endogenous monocyte *IL1B*. With regard to the LPS-unresponsive state known as endotoxin tolerance, our data revealed that following transient induction, *IL1B* and *TNF* remained marked with paused Pol II complexes for up to 24 hours post-stimulation. Upon subsequent LPS exposure, tolerized *TNF* remained in an unresponsive paused state, while *IL1B* resumed transcription due to recruitment of the positive elongation kinase P-TEFb. Emerging evidence suggests that inflammatory responses of LPS/TLR4 activated macrophages are interconnected with metabolic pathways, resulting in the shift of energy utilization by the cells [15]. Here we report that inhibition of either phosphoinositide 3-kinase (PI3K) or glucose uptake had a greater affect on the transcriptional response of *IL1B* than of *TNF*. The differences between these two genes, especially for endotoxin tolerance, suggest that *IL1B* may play a distinct role from *TNF* in chronic inflammation. It should also be noted that the gene nomenclature in this paper varies with species. Specifically, human genes are represented by all uppercase gene designations (e.g., *IL1B*, *TNF*), whereas mouse loci are designated by the capitalization of only the first letter (*Il1b*, *Tnf*).

## Results

### *IL1B* and *TNF* mRNA are Differentially Expressed in Monocytes

**Figure 1:**
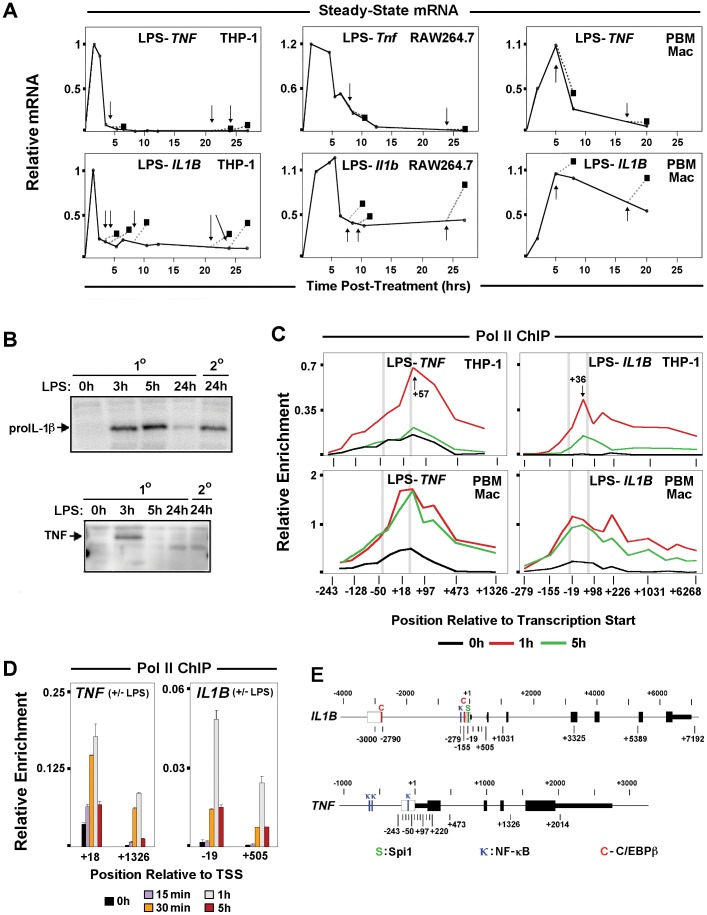
Comparison of *IL1B* and *TNF* expression in monocytes. (A) Steady-state mRNA kinetics for *IL1B* and *TNF* transcripts in LPS stimulated THP-1, RAW264.7 and human peripheral blood mononuclear cells (hPBMC). Solid lines denote mRNA levels for primary LPS challenge. Squares show transcript levels following re-stimulation, as indicated by arrows. (B) Western blot for 30 KDa proIL-1β precursor protein. (C) Pol II ChIP throughout the *IL1B* and *TNF* loci in resting (black), 1 h (red) and 5 h (green) LPS stimulated THP-1, RAW264.7 and hPBMC cells. Vertical gray bars locate the positions of important gene landmarks. These include TATA box and the canonical Pol II pause position (approximately 30 bp upstream and 50 bp downsteam of TSS, respectively). (D) Pol II ChIP at promoter and downstream sites for *IL1B* and *TNF*. E. Schematic of *IL1B* and *TNF* gene structures showing exons (solid boxes), positions of ChIP amplicons (midpoint relative to TSS), and important transcription factor binding sites (**C**: C/EBPβ, **κ**: NF-κB and **S**: Spi1) within regulatory regions (open boxes).

Steady-state mRNA kinetics of *IL1B* and *TNF* in human (THP-1) and murine (RAW264.7) monocyte cell lines, as well as primary macrophages, revealed differences in transcription responses. *TNF* displayed rapid induction and complete transcription shut down within a few hours of LPS treatment. In contrast, *IL1B* was also rapidly induced, but not completely switched off, with continued expression for many hours post-stimulation (Figures 1A, S1A–C). Transient expression patterns for these genes are reflective of their transcription because of short mRNA half-life mediated by AU-rich element (ARE) degradation, especially during the first 5 h after induction for *IL1B* message, as reported for THP-1 cells [3], [16]. In resting monocytes, basal levels of full-length unspliced *TNF,* but not *IL1B,* transcripts were detected (Figure S1D). It has been hypothesized that low levels of constitutive transcription favors accessible chromatin and transcription competence for IE gene activation [17]. RNA polymerase II (Pol II) ChIP-qPCR was used in order to directly measure the transcription status of monocyte *IL1B* and *TNF* (Figures 1C, S2A). Pol II occupancy kinetics, particularly in THP-1 cells (Figure 1C), mimicked the respective steady-state mRNA profiles confirming that sustained expression of *IL1B* shown in Figure 1A resulted from continuous polymerase engagement and not from increased mRNA stabilization. Kinetic analysis of the transient phase of THP-1 gene activation revealed a 30 minute delay in Pol II recruitment to *IL1B* (Figure 1D), consistent with the observed mRNA delay (Figure S1E). In contrast, increased Pol II binding on *TNF* was detected as early as 15 min following LPS stimulus. We next asked, whether the differential shutdown of these genes corresponded to differences in tolerance after exposure to secondary LPS stimulus. We observed that both mouse and human genes coding for TNFα were tolerized, so that once induced they could not be re-stimulated. A previous report argued that murine *Ilib* and *Tnf* are both refractory to reactivation due to mechanisms commonly recognized as endotoxin tolerance [2]. In contrast to that report, in which cells were washed prior to re-stimulation, we observed significant transcription of genes coding for IL-1β after repeated LPS exposure in THP-1, RAW264.7, and human primary macrophages stimulated with LPS followed by an equal 2.5 h secondary dose added to unwashed cultures (Figure 1A, arrows, boxes and dotted lines). Western blot analysis demonstrated that secondary stimulation of *IL1B* resulted in expression of the 31 KDa proIL-1β precursor protein, but not the 26 KDa precursor for TNFα (Figure 1B). Strikingly, these results recapitulate an earlier report that *in vivo* injection of a sub-lethal dose of LPS into mice resulted in TNF, but not IL-1 tolerance in serum [4]. Similarly, steady-state kinetic mRNA secondary stimulation revealed that *IL1B* transcription is not tolerized (Figure 1A).

### Pol II Pausing and the P-TEFb:NELF Axis Contribute to Differential Transcription Shutdown of *IL1B* and *TNF*

**Figure 2:**
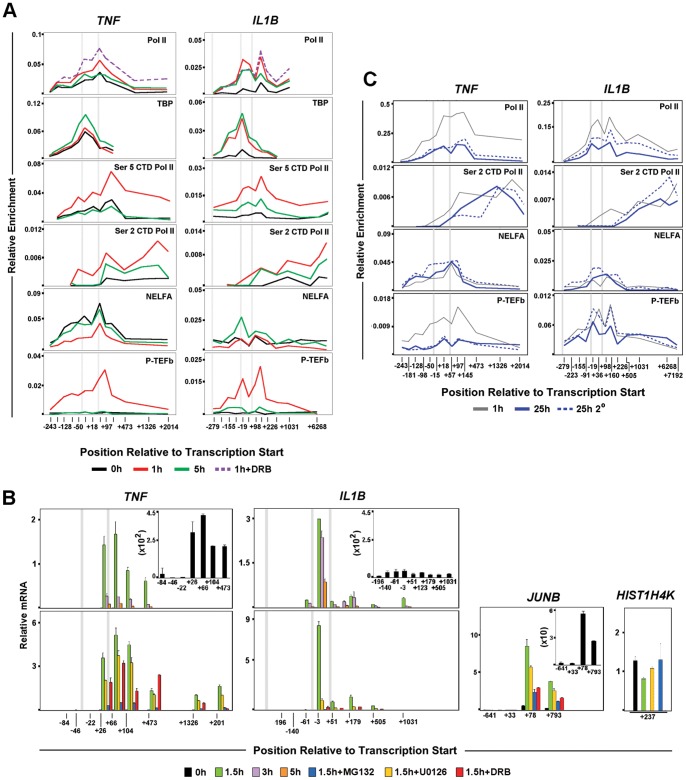
Distribution of factors relevant to differential transcription regulation and endotoxin tolerance for *IL1B*. (A) ChIP for factors related to Pol II elongation at *IL1B* and *TNF* loci in THP-1 cells. (B) Steady-state mRNA kinetic positional profiles for *IL1B*, *TNF* and control gene transcripts, as indicated, in LPS stimulated THP-1. (C) ChIP for *IL1B* and *TNF* during secondary LPS stimulation of THP-1 cells. The solid and dotted plots represent primary and secondary LPS treatment, respectively, of THP-1 cells at indicated times. Thin gray plot denotes 1 h LPS reference curve. For all panels, along with the two gene landmarks in Figure 1C, an additional vertical gray bar designates the location of an important NF-κB binding site (near −300 bp) for *IL1B*.

IE gene activation associated with signal dependent release of pre-loaded Pol II facilitates rapid gene transcription [18], [19]. Since *IL1B* and *TNF* are immediately transcribed in activated monocytes, promoter Pol II enrichment was examined. *TNF* exhibited a significant Pol II peak (centered at +57), downstream of the transcription start site (TSS) in resting THP-1 and human primary macrophages (Figure 1C). A significant amount of paused Pol II (centered at +36) at *IL1B* was prominent only in LPS stimulated cells (Figures 1C, S2A, S2B). Consistent with elongation, LPS activation caused increased Pol II throughout transcribed regions of both genes. Differential TBP binding between *IL1B* and *TNF* in resting and induced cells, supports differential Pol II pre-association for these genes (Figures 2A, S2A). The sites of paused Pol II were associated with inducible short transcripts (Figure 2B, upper panels) sensitive to the specific transcription factor inhibitors U0126 for C/EBPβ and MG132 for NF-κB (Figure 2B, lower panels). **While MG132 blocks proteosome degradation of the inhibitory IκB protein [20], U0126 blocks the activation of ERK/MAPK phosphorylation pathway [21], [22]**. In agreement with Pol II ChIP, basal *TNF* transcription in unstimulated monocytes was further confirmed with this technique. *TNF* data closely resembled that of classically paused *JunB* (Figure S2C). Control *HIST1H4K* gene transcript amplification was constitutively expressed (Figure 2B). Pol II dynamics for murine RAW264.7 cells and bone marrow derived macrophages (BMDM) confirmed similar differences between *Il1b* and *Tnf* (Figure S3A,B).

Phosphorylation of Pol II carboxy-terminal domain (CTD) and recruitment of cofactors, such as NELF and P-TEFb are important indicators of initiation, pausing and elongation [23]. Figure 2A shows kinetics for indicators of transcription elongation for LPS-treated THP-1 cells. As expected, enrichment of Pol II S5P CTD is confined to promoter proximal regions, whereas S2P CTD in LPS-stimulated monoctyes increased toward the 3′ end of both genes. Increased binding of NELF to paused Pol II on *TNF* was diminished within 1 h. As transcription concludes, around 5 h post stimulation, NELF binding to *TNF* returned to pre-stimulation levels. NELF ChIP for *IL1B* revealed distinct binding with increased enrichment at later time points, confirming the lack of a Pol II pause in resting monocytes. P-TEFb was coordinately recruited to the promoters of both genes, but in contrast to *TNF,* its binding was maintained on *IL1B* at 5 hours in THP-1 cells, although at a lower level than at 1 h (Figure 2A) and significantly prolonged in BMDM (Figure S3B) and likely contributed to sustained expression. P-TEFb inhibitor 5,6-dichloro-1-beta-D-ribofuranosylbenzimidazol (DRB) blocked transcription and maintained Pol II at the proposed paused sites (Figure 2A,B), demonstrating the significance of P-TEFb in inducible control of *IL1B* and *TNF*.

### Pol II S2P CTD Differentially Influences *IL1B* and *TNF* Endotoxin Tolerance

ChIP revealed a significant amount of promoter Pol II on both genes in 25 h stimulated monocytes, with decreased signal downstream of the pause sites (Figure 2C). Pol II occupancy within the gene was slightly higher for *IL1B*, likely explaining its sustained transcription profile. NELF was co-localized with promoter bound Pol II on both genes (Figure 2C). Our data revealed that upon secondary stimulation, P-TEFb was re-recruited to the *IL1B* promoter, resulting in resumption of elongation. This is in contrast to tolerized *TNF*, in which P-TEFb recruitment and S2P CTD levels were not increased in re-stimulated cells (Figure 2C). The results suggest that low levels of sustained expression may sufficiently maintain *IL1B* competency for secondary re-induction by the means of gene-specific liberation of the Pol II pause by P-TEFb during repeated LPS exposure, a situation that fails to occur on *TNF*. These data argue that secondary induction of *IL1B* is a physiologically significant phenomenon that further distinguishes it from *TNF*.

### LPS Stimulation of Monocytes Results in Dynamic Changes in Nucleosome Positioning and Modification on *IL1B* and *TNF*

**Figure 3:**
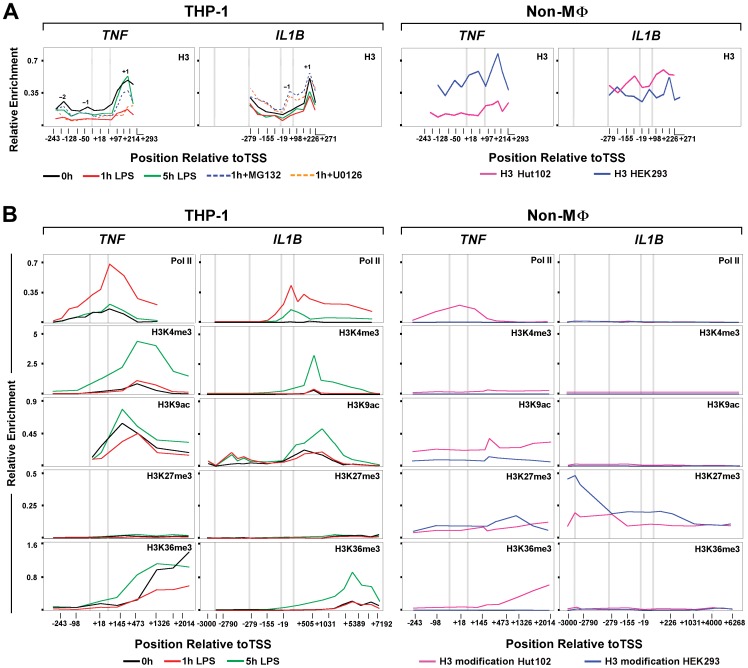
Nucleosome positioning dynamics and modifications during *IL1B* and *TNF* induction. (A) Kinetic ChIP of histone 3 (H3) for *IL1B* and *TNF* in THP-1 and control Hut102 and HEK293 cells, as indicated. Key nucleosomes are designated by position relative to the TSS (−2, −1, +1). (B) ChIP for histone modifications at *IL1B* and *TNF*, as indicated for each cell line. All panels are similarly scaled with respect to spatial distribution along each gene, permitting comparative localization. For all panels, along with the three gene landmarks in Figure 2, an additional vertical gray bar designates the approximate location of the far-upstream enhancer (−3000 bp) for *IL1B*.

Nucleosome position plays a critical role in promoter accessibility, and genome-wide studies have shown that *Drosophila* and human promoters are commonly devoid of nucleosomes [24], [25]. Stalled Pol II serves as a physical barrier, preventing promoter nucleosome assembly and formation of repressive chromatin, enabling gene expression [26]. To address the question of chromatin influence on these two genes, promoter nucleosome occupancy was examined using core histone 3 (H3) ChIP [24], [27] in resting and stimulated THP-1 monocytes, HEK293 pre-neuronal cells that express neither *IL1B* nor *TNF*, and HUT102 cutaneous T lymphocytes that constitutively express *TNF* (Figure 3A). We observed +1 nucleosomes on both genes approximately 200 bp downstream of TSS. A similar observation was reported for *Drosophila Hsp70* promoter [28]. The distribution of weakly positioned nucleosomes, upstream of TSS, was unique to each gene. In particular, the −1 nucleosome on *TNF* was located approximately 40 bp upsteam of the TSS, whereas on *IL1B* it was focused further downstream in the vicinity of the TSS. We observed a significant depletion of promoter bound nucleosomes in 1 h-stimulated monocytes, similar to that reported for activated genes [29]. The extent of nucleosome depletion was reduced in cells pretreated with inhibitors U0126 and MG132 selective for transcription factors associated with induction of one or both genes (Figures 3A). Therefore, this depletion is stimulation dependent, requiring specific factor recruitment. It is noteworthy that *IL1B* nucleosome displacement was sensitive to both inhibitors, whereas *TNF* was almost exclusively affected by MG132, suggesting that C/EBPβ is specifically required for *IL1B*. Five hours post-stimulation, as Pol II recruitment levels decline, depleted nucleosomes recovered, approaching initial enrichment levels for the +1 nucleosome of *TNF*. In contrast, *IL1B* nucleosome depletion exhibited only a partial recovery. In addition, cells pre-treated with either inhibitor revealed a striking increase in the −1 *IL1B* nucleosome, an additional distinction from *TNF*. The presence of uniquely phased −1 nucleosomes in promoter NDR has been suggested to inhibit Pol II recruitment [30], [31], but to our knowledge this is the first report indicating its role affecting inducible IE activation in human immune cells and may reflect loss of an important priming function for this gene. The *IL1B* and *TNF* nucleosomes in HEK293 exhibited higher levels, especially for the −1 nucleosome. Nucleosomes were similarly more abundant on *IL1B* in Hut102 than in THP-1, with higher levels at −2 and +1. The constitutive expression of *TNF* in Hut102 revealed a profile similar to that for 1 h stimulated THP-1 cells (Figure 3A).

To further understand the processes regulating IE gene architecture and LPS induction, the spatial-temporal distribution of chromatin marks on *IL1B* and *TNF* was investigated (Figure 3B, S4). LPS caused changes in nucleosome marks on both genes. We observed the absence of repressive H3K27me3 and high levels of permissive H3K4me3 in monocytes that likely contributes to LPS responsiveness [32]. H3K4me3, a mark of promoter regulatory elements that indirectly facilitates TBP recruitment [33], did not show a significant 1 h post-LPS increase in the vicinity of +1 nucleosomes. Surprisingly, enrichment of this mark revealed delayed kinetics on IL1B and followed Pol II recruitment, as shown by an increase at 5 h. Higher levels of H3K4me3 at 5 h remained mostly at the *IL1B* promoter, but spread throughout the body of the *TNF* gene. The distinct positional effect of H3K4me3 near the promoter versus the downstream region of genes has previously been observed [34], and may be critical for differences between *IL1B* and *TNF*. The relative levels of the activating H3K9ac mark on the +1 nucleosome and of heterochromatic H3K9me3 on downstream nucleosomes within the gene body, suggest a possible relationship with post-stimulatory tolerance for *TNF*. Prior to stimulation both genes were associated with permissive levels of H3K9ac at the +1 nucleosome, supporting gene expression competency. Importantly, H3K9ac levels were initially very low in the vicinity of the *IL1B* upstream enhancers, but were significantly increased at 1 and 5 h post-stimulation. Examination of nucleosome marks at the promoters for these genes (Figure 3B) normalized to the relative H3 levels on nucleosome +1 at each time point (Figure 3, Table S1 in File S1), suggest that the significant levels of H3K9ac on +1 did not appear to significantly increase in either gene at 1 h post-stimulation. For *IL1B,* the −1 nucleosome, in contrast to that at +1, revealed at least a 10-fold increase in H3K9ac by 1 h that persisted at 5 h post-stimulation. In contrast to *IL1B*, 5 h post-stimulated *TNF* uniquely exhibited a large increase in H3K4me3 throughout the gene body, with a lesser increase than *IL1B* in H3K9ac at the +1 nucleosome. At 5 h H3K4me3 is increased more selectively over the +1 nucleosome along with H3K9ac. It is possible that this distinction maintains the sustained elongation and resistance to tolerance for *IL1B*. High levels of the H3K9me1 promoter-proximal mark were distributed throughout the transcribed gene body of *IL1B* and *TNF* in resting monocytes, a phenomenon reported by others with unknown functional significance [35]. In contrast to H3K9ac, the H3K9me1 was rapidly lost following LPS treatment (Figure S4). The high levels of H3K9me1 in the non-monocytic cell lines suggest that this mark might contribute to gene suppression when present at sites distal from the TSS. We hypothesized that the TLR4-dependent activation of *IL1B* and *TNF* caused replacement of the repressive H3K9me1 mark with a transcriptionally permissive one, which may have contributed to the expression of both genes.

The Pol II elongation footprint marked by H3K36me3 [36] revealed a consistent LPS-induced transient enrichment pattern that increased toward the 3′ ends of both genes. In contrast to *IL1B*, significant levels of H3K36me3 were detected on the *TNF* locus in unstimulated monocytes, further confirming constitutive basal activity. The spatial distribution of chromatin modifications at *IL1B* and *TNF* loci were also assessed for Hut102 and HEK293 cells. Pol II levels and chromatin marks for *TNF* in Hut102 were consistent with active transcription, as previously reported [37], whereas *IL1B* was repressed (Figure 3B). *TNF* in Hut102 revealed low, but significant, levels of promoter-proximal H3K4me3, and moderate levels of H3K27me3. This combination is a “bivalent” mark [32], indicative of inactive/poised developmental induction. Nevertheless, TNF in these HTLV-1 infected malignant T cells is constitutively expressed, suggesting a more complex means of gene regulation. HEK293 did not show positive indicators for either gene and exhibited only the inhibitory H3K27me3 on both. The repressive H3K27me3 extended throughout the length of both genes, but appeared to be more focused over the *TNF* gene body, while for *IL1B* the mark was more prominent over the potent LPS enhancer near −3000, which binds C/EBPβ [38], and the key NF-κB site near −300 [10].

### Spi1 Mediates Monocyte-specific *IL1B* Expression

**Figure 4:**
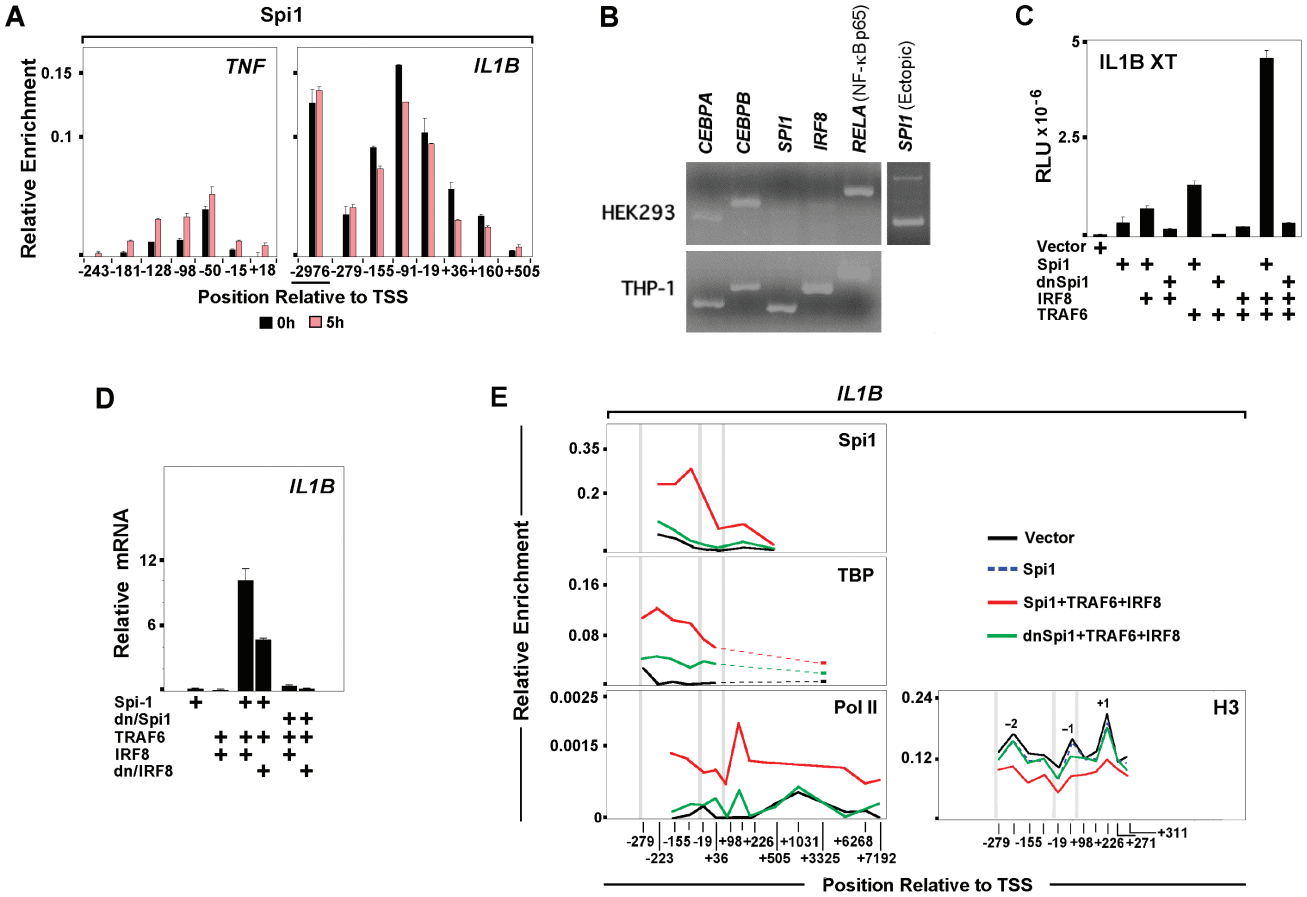
Spi1 mediates monocyte-specific *IL1B* expression. (A) Spi1 ChIP for *IL1B* and *TNF* in control and LPS-treated THP-1 cells. (B) Transcription factor mRNA expression profiles in HEK293 and THP-1 cells. A third panel (and data in Figure S5H) displays ectopic mRNA expression of Spi1 in transfected HEK293. (C) IL1BXT-Luc reporter activity for ectopic expression of indicated factors in HEK293. (D) Endogenous *IL1B* mRNA expression in transfected HEK293. (E) ChIP for endogenous TBP, Pol II and H3 with ectopic Spi1 in HEK293. Vertical gray bars designating important gene landmarks are as described in Figure 3.

Evaluation of the spatial-temporal distribution of selected transcription factors revealed that *IL1B* is dependent upon a different set of regulators than *TNF*. A major factor involved in genome-wide priming of LPS responsive enhancers [39], [40] and maintenance of the macrophage lineage is the ETS domain DNA binding factor Spi1 [41]. We previously reported that vigorous *IL1B* transcription depends on Spi1 binding both to the *IL1B* promoter [9] and to a poised monocyte-specific enhancer, requiring cooperative association of Interferon regulatory factor 8 (IRF8) and non-tyrosine phosphorylated (NTP)-Stat1 [42]. In agreement with this, constitutive association of Spi1 at the *IL1B* promoter and enhancer persisted for an extended time post induction (Figure 4A). In contrast, Spi1 was significantly less abundant on *TNF* (Figure 4A). We hypothesized that in addition to its role priming enhancers, Spi1 binding at the *IL1B* promoter mediates cell-type restricted transcription competency. To examine the role of this “pioneer factor” in *IL1B* induction, transient transfection studies were carried out in HEK293 cells, which do not transcribe *IL1B*. Initial screens revealed the absence of Spi1 in these cells as compared to THP-1 (Figure 4B). Since HEK293 do not express the TLR4 LPS receptor, co-transfection of TNF receptor-associated factor 6 (TRAF6) was used as a dominant-positive LPS surrogate in these cells [43]. Figure 4C shows that an *IL1B* reporter vector (XT-Luc) was potently up-regulated by Spi1 in combination with IRF8, a factor important for full *IL1B* activity in monocytes [42] and absent in HEK293, and dominant-positive TRAF6. IRF8 and TRAF6 alone (Figure S5A) or in combination were insufficient for *IL1B* induction. Spi1 function requires the integrity of its N-terminal TBP Binding Domain (TBD) [9]. In agreement, ectopic expression of a dominant-negative Spi1 mutant (dn/Spi1), containing only the Spi1 DNA binding domain, reduced XT-Luc activity to background levels. Analysis of endogenous *IL1B* mRNA in HEK293 transfected with the same factors supported the luciferase assay results as well as the critical role of Spi1 for *IL1B* induction (Figure 4D). Basal level of *IL1B* transcription in cells transfected only with Spi1 was increased by addition of IRF8 and TRAF6. Substitution of wild type with dn/Spi1 abolished *IL1B* expression. *TNF* expression in Spi1 transfected HEK293 was unaffected (Figure S5B.). Since the N-terminal TBD of Spi1 directly interacts with TBP [5], we tested whether Spi1 plays a role in recruitment of TBP to the *IL1B* promoter by performing ChIP in HEK293 transfected with either wild type or dn/Spi1 in combination with IRF8 and TRAF6. As shown in Figure 4E, transfection of Spi1 and the auxiliary factors increased TBP occupancy at the *IL1B* TATA box. We observed recruitment of Pol II to *IL1B* downstream of TSS, reminiscent of paused polymerase, as well as to the transcribed region of the gene, consistent with elongation. TBP and Pol II occupancy in HEK293 transfected with dn/Spi1 were dramatically reduced. Transfection-induced *IL1B* activation was also associated with depletion of promoter proximally phased nucleosomes. Figure 4E shows that full length Spi1 in combination with TRAF6 and IRF8 were necessary for *IL1B* promoter nucleosome depletion. These data suggest that Spi1 plays a critical role at the *IL1B*, but not the *TNF* promoter. In addition to facilitating *IL1B* promoter accessibility [44], the Spi1 TBD may play a role in general transcription machinery recruitment by TBP.

### LPS-activated C/EBPβ Interaction with Spi1 Differentiates Induction of *IL1B* and *TNF*

**Figure 5:**
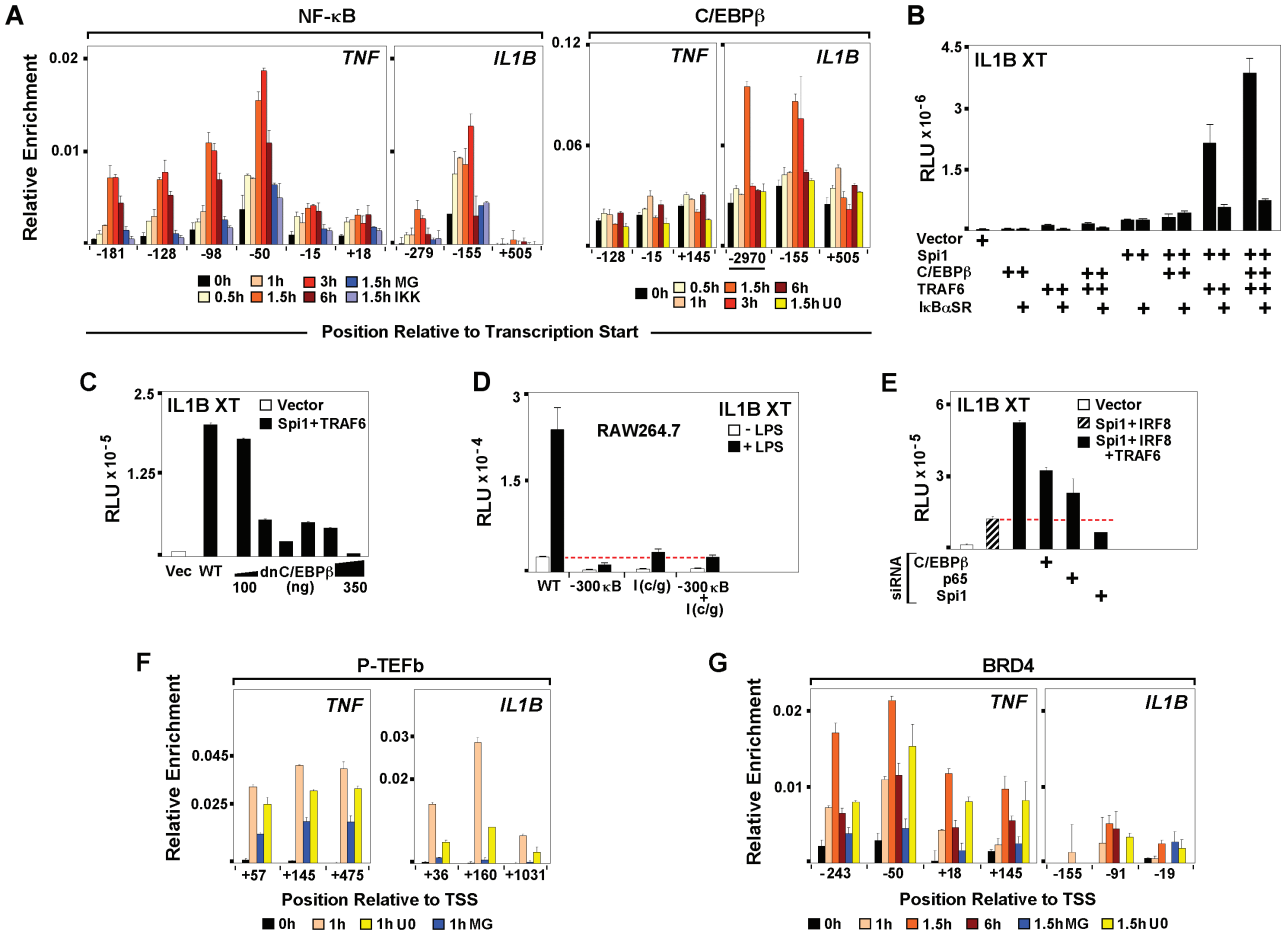
LPS-activated C/EBPβ interaction with Spi1 differentiates induction of *IL1B* and *TNF*. (A) NF-κB and C/EBPβ ChIP for THP-1 cells, as indicated. (B) Effect of ectopic expression of IκBα super repressor (IκBαSR) on IL1B XT-Luc reporter activity in HEK293 cotransfected with indicated factors. (C) Effect of dnC/EBPβ titration on IL1B XT-Luc reporter activity in HEK293. (D) Effect of C/EBPβ and NF-κB binding site mutations on IL1BXT-Luc reporter activity in RAW264.7. (E) XT-Luc reporter activity, as indicated in HEK293 cotransfected with C/EBPβ, NF-κB and Spi1 siRNA. (F) P-TEFb and (G) BRD4 ChIP in THP treated, as indicated.

Endotoxin dependent binding of NF-κB has been shown to play an important role during induction of *IL1B* and *TNF* in monocytes [10], [45] and Spi1 may facilitate NDR formation by exposing binding sites for LPS-responsive transcription factors [46]. Kinetic ChIP analyses revealed transient binding of NF-κB to both genes within 30 minutes of LPS treatment, which was diminished by pre-treatment of THP-1 with either the NF-κB-targeted proteasome inhibitor MG132 or the IκB kinase inhibitor BMS-345541 (Figure 5A). Concomitantly, mRNA levels of both genes were significantly reduced (Figure S5C–E). Earlier *in vitro* studies demonstrated the involvement of C/EBPβ in *IL1B* induction [8], [47]. Using ChIP to evaluate *in vivo* binding kinetics for C/EBPβ in LPS-stimulated cells revealed LPS-mediated recruitment of C/EBPβ to the *IL1B,* but not to the *TNF* promoter (Figure 5A). The U0126 MEK1/2 pathway inhibitor was chosen in order to target the activation of C/EBPβ. LPS activated monocytes pre-treated with U0126 revealed decreased *IL1B* transcription (Figure S5E), consistent with reduced C/EBPβ binding (Figure 5A), while *TNF* expression was unaffected. Transient transfection of HEK293 was used to clarify the role of these inducible transcription factors. NF-κB and C/EBPβ were ineffective *IL1B* inducers when transfected alone into HEK293, but significant activation of *IL1B* was observed when the factors were transfected in combination with Spi1 (Figures 5B, S5F). Co-expression with TRAF6 showed the strongest activity (Figure S5F). Over-expression of an IκBα super repressor (IκBαSR), [48] considerably reduced, but did not completely abolish, *IL1B* activity in HEK293 transfected with Spi1, TRAF6 and C/EBPβ (Figure 5B). Experiments in murine RAW264.7 monocytes further demonstrated that IκBαSR fully eliminated NF-κB activity without completely inactivating *IL1B* XT-Luc (Figure S5G). In addition, titration of truncated, dn/C/EBPβ [8] in HEK293, confirmed dose dependent inhibition of *IL1B* activity (Figure 5C). To further demonstrate the importance of NF-κB and C/EBPβ for *IL1B* induction, RAW264.7 cells were transiently transfected with a modified *IL1B* XT-Luc reporter harboring mutations within the essential NF-κB (near −300) and C/EBPβ (I-Region/Enhancer) binding sites. As shown in Figure 5D, disrupted binding of these two factors severely reduced responsiveness of *IL1B* reporter to LPS. Lastly, siRNA for NF-κB and C/EBPβ in HEK293 revealed significant reduction of *IL1B* XT-Luc activity (Figure 5E). In agreement with our previous results, depletion of Spi1 caused severe reduction of *IL1B* reporter activity (Figure 5E). The data presented here, challenge the popular notion that NF-κB is the only critical LPS-activated factor potently affecting *IL1B* induction. It appears that NF-κB and C/EBPβ cooperatively regulate LPS induced transcription of *IL1B*, while expression of *TNF* appears to be influenced primarily by NF-κB.

We next explored the relationship between the factors and dynamics of paused Pol II release during transcription of *IL1B* and *TNF* by examining P-TEFb ChIP for LPS stimulated THP-1 cells pre-treated with specific transcription factor inhibitors. Inhibition of NF-κB activation by MG132 resulted in significant depletion of P-TEFb recruitment to both genes (Figure 5F). This is consistent with reports that NF-κB interacts with and activates P-TEFb [49]. There was also a rapid-transient recruitment of the bromodomain-containing protein BRD4 to *TNF* within 30 minutes of LPS stimulation, whereas occupancy of BRD4 on *IL1B* was less prominent (Figure 5G). BRD4 is an atypical kinase reported to phosphorylate Pol II S2 CTD [50], which is recruited to NF-κB p65 phosphorylated at Ser 276 by MSK-1 MAP kinase [49]. These results suggests a possible novel role for C/EBPβ as an adaptor, mediating the recruitment of P-TEFb to the *IL1B* promoter. As expected, only minor changes in P-TEFb occupancy at the *TNF* promoter were observed in U0126 exposed cells.

### Transcription Factor Mediated Looping between the *IL1B* Distal Enhancer and Promoter

**Figure 6:**
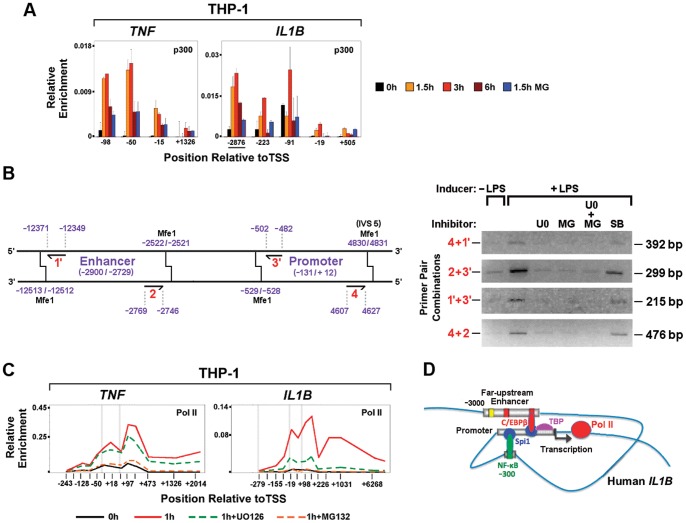
LPS-dependent p300 binding and transcription factor-mediated promoter-enhancer looping at *IL1B*. (A) Inducible p300 binding at *IL1B* and *TNF*. **(**B) Schematic representation of PCR primer pairs used for evaluating 3C ligation products (Left) and PCR assessment of 3C ligation restriction fragment products in the absence and presence of U0126 and MG132 inhibitors. (C) Effects of U0126 and MG132 inhibitors on Pol II ChIP for *IL1B* and *TNF*, as indicated. (D) Model for chromatin looping during activation of *IL1B*.

Previous reports have identified far-upstream enhancers, positioned −3000 bp from the TSS for human *IL1B* and −2200 for mouse *Ilib*, as critical for robust induction [38], [51]. Recent genome-wide studies in murine macrophages demonstrate that LPS responsive enhancers have common features marked by inducible p300 binding and H3K4me1 modification [39], [40]. Our analysis of H3K4me1 revealed significant enrichment of this mark throughout the transcribed regions of *IL1B* and *TNF*, as well as at the −3000 bp far-upstream *IL1B* enhancer (Figure S4). In agreement with robust LPS-mediated p300 binding (Figure 6A), we observed that H3K9 acetylation levels increased throughout the enhancer in LPS stimulated monocytes (Figure 3B). Chromosomal interactions between distal elements have been implicated in regulating gene expression [52]. The dynamic association of enhancers and promoters can be mediated by protein-protein and protein-DNA or RNA interactions among transcription factors and chromatin modifiers, ultimately leading to enhanced initiation [53]. On the basis of *in vitro* studies, functional cooperation between enhancer bound C/EBPβ and promoter bound Spi1 DNA looping was previously proposed as a mechanism for *IL1B* induction [14], [54]. We used chromosome conformation capture (3C) to examine LPS-dependent *in vivo* long-range chromosomal interactions between the *IL1B* enhancer and promoter. Figure 6B reveals LPS-dependent physical association between *IL1B* distal and proximal regulatory elements. The NF-κB and C/EBPβ inhibitors abolished LPS dependent chromosome loop formation (Figure 6B), transcription (Figure S5E), nucleosome depletion (Figure 3A) and Pol II recruitment to the *IL1B* promoter (Figure 6C), revealing that chromosome looping correlates with the binding of C/EBPβ and NF-κB. In addition to interacting with C/EBPβ, the DNA binding domain of Spi1 interacts *in vitro* with NF-κB (Figure S5I). These data suggest that endotoxin activation of both C/EBPβ and NF-κB may contribute to dynamic juxtapositioning of the distal regulatory elements of *IL1B* by common association with two critical Spi1 binding sites previously mapped to the IL1B promoter [9], resulting in the formation of a chromatin complex favorable for gene induction (Figure 6D).

### Metabolic Effects on Transcription Regulation of *Il1b* and *Tnf*

**Figure 7:**
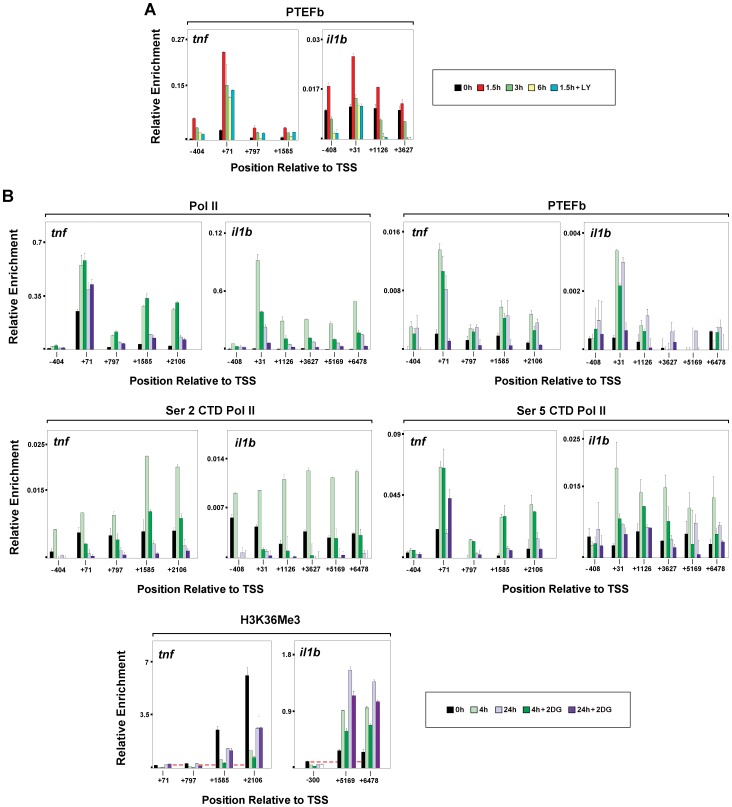
Distinct metabolic sensitivity for transcription elongation on *Il1b* and *Tnf* in murine bone marrow-derived monocytes. (A) P-TEFb ChIP for mouse RAW264.7 *Il1b* and *Tnf* genes in the presence of LY-294002 inhibition. (B) Pol II, PTEFb, S2P CTD Pol II, S2P CTD Pol II and H3K36me3 ChIP, as indicated, for 2DG-treated mouse BMDM. The BMDM were stimulated for indicated times with LPS plus or minus 3 h pretreatment with 2–DG.

Since P-TEFb recruitment to *IL1B*, in contrast to *TNF*, appeared to be less dependent upon BRD4 and more dependent upon C/EBPβ, distinct pathways for P-TEFb activation by release from the inhibitory 7SK RNP complex [1] were considered. One of these is the possible involvement of PI3K as an activator of Akt/PKB kinase, which has been reported to activate P-TEFb by directly phosphorylating Hexim1 in 7SK RNP [55]. Figure 7A reveals that the PI3K inhibitor LY-294002 had a greater effect on P-TEFb recruitment to *Il1b* than to *Tnf* in LPS-treated RAW264.7 cells. Interestingly, ligand-mediated activation of both TLR and IL-1 receptors not only induces *IL1B* transcription, but also directly recruits and activates PI3K [56], [57], consistent with the proposed role for PI3K and Akt in P-TEFb activated induction. This result was of particular interest to us because we have recently reported that the non-metabolizable glucose analogue and hexokinase inhibitor 2-deoxyglucose (2-DG) [58], which directly inhibits glycolysis and ATP synthesis, more effectively inhibits *IL1B* than *TNF* in a manner that is dependent upon the stabilization of the HIF-1α transcription factor binding to *IL1B* under normoxia conditions approximately 4 h after LPS induction [59]. Figure 7B reveals the presence of significantly higher levels of Pol II and Pol II S5P CTD on *Tnf* than on *Il1b* for 4 h LPS stimulated murine BMDM pretreated with 2-DG. This result is consistent with reduced levels of Pol II S2P CTD, P-TEFb and H3K36me3 relative to Pol II on *Il1b*. (Figure 7B). Overall, the greater sensitivity of *Il1b* elongation to the metabolic state of the cell may position P-TEFb as a critical regulator of inflammatory responses.

## Discussion

**Figure 8:**
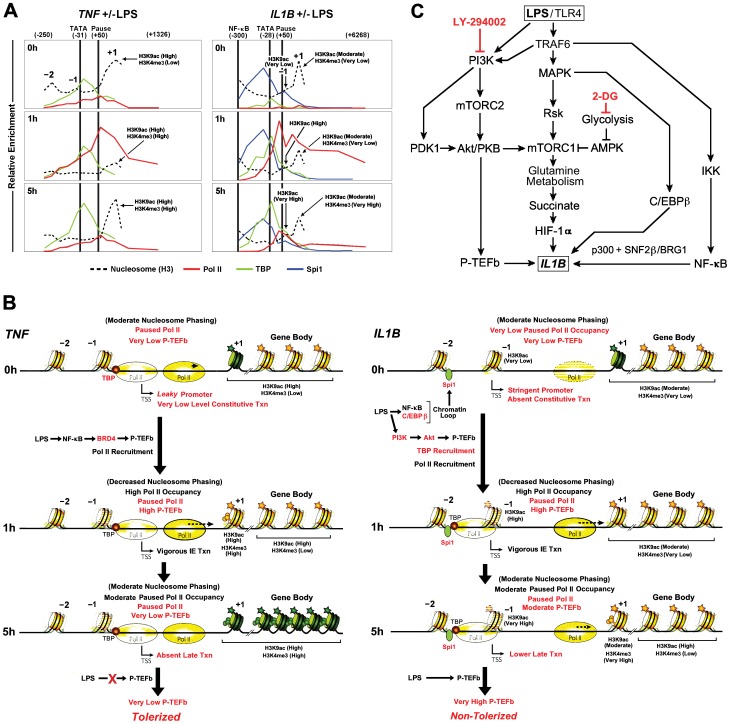
Proposed mechanism for LPS mediates induction of *IL1B* and *TNF* in monocytes. (A) Summary of ChIP kinetics for some key features of *IL1B* and *TNF* in THP-1 monocytes. Pol II, TBP and Spi1 are as indicated. Histone modifications at specific locations detailed in the text are labeled. Key nucleosomes are designated by position relative to the TSS (−2, −1, +1). (B) Models for *IL1B* and *TNF* gene regulation. Red text highlights important distinctions between the two genes along the induction kinetic. Nucleosomes are marked with stars (acetylation) and spheres (trimethylation) representative of significant increases in modification. Darkly colored nucleosomes are likely to be less dynamic and suggestive of impediments to gene expression. The indicated locations of Pol II are represented by various levels of intensity, reflecting the relative degree of proposed dwelling on DNA. Arrowheads on Pol II represent the relative efficiency of elongation, as indicated by the length of the associated dotted line. (C) Schematic representation of the relationships between metabolic pathways involved in *IL1B* gene activation, summarizing key elements from this study and that recently reported elsewhere [56].

The induction of *IL1B* and *TNF* involves a stringently regulated sequence of events triggered by the detection of LPS by TLR4. Previous reports suggest that IE gene activation is associated with a pre-assembled promoter transcription machinery [17], [60]. Paused Pol II and a depleted nucleosome architecture favors the immediate response to stimuli by transitioning into a state of processive elongation [61]. Although *IL1B* and *TNF* are both classified as TLR4-dependent IE genes, we observed that their transcription was differentially regulated in LPS treated monocytes. Our detailed kinetic analysis of transient *vs*. sustained expression provides novel insight into changes associated with induction, shutdown, and the potential for reactivation of these genes. In particular, changes in transcription factor recruitment and nucleosome occupancy may all contribute to the rapid gene-specific induction of these IE responders. Figure 8A summarizes some of the relevant data, along with a detailed model in Figure 8B. In unstimulated cells, the *TNF* promoter contains significant pre-bound TBP and NELF-dependent paused Pol II. The pre-assembled components of the transcription machine likely contribute to the observed constitutive transcription *leakiness* of *TNF*, priming it for rapid induction. Therefore, *TNF* fits the current models for IE gene induction involving prebound TBP [62] and paused Pol II. Quiescent *IL1B* is initially more stringently regulated, recruiting very low levels of TBP and Pol II. The initial induction of *IL1B* is primarily dependent upon LPS-dependent Pol II recruitment, followed secondarily by a transient DRB-sensitive and p-TEFb-dependent Pol II pause. The Pol II peaks on *TNF* and *IL1B* are associated with short nascent transcripts whose levels correlate with temporal binding of enzyme and inhibitor sensitivity. Phosphorylation of NELF and S2P CTD, mediated by P-TEFb, transitions Pol II to elongation. The slower initial expression of *IL1B* as compared to *TNF* correlates with a similar delay in Pol II recruitment, arguing for an upstream rate-limiting step, which could be related to the *de novo* recruitment of TBP and Pol II. Interestingly, the 3C results, demonstrating the existence of a chromatin loop, consistently revealed the prevalence of one recombination product, suggesting the possibility of a preferred conformational proximity for the upstream and downstream *IL1B* sequences. Such preformed chromatin architecture has been observed for cells at specific developmental stages [63]. Here we show that LPS-inducible binding of NF-κB to *TNF* facilitates recruitment of BRD4, and subsequently P-TEFb, consistent with previous studies of murine macrophages [17]. In contrast to *TNF*, both NF-κB and C/EBPβ appear to mediate BRD4-independent recruitment of P-TEFb to *IL1B* (Figure 5F,G). Additionally, we argue that PI3K and Akt-mediated activation of P-TEFb selectively contributes to *Il1b* elongation in murine macrophages. Since metabolic imbalance affects PI3K/Akt signaling, a disruption of glucose availability in stimulated monocytes may cause selective inhibition of Pol II elongation on *Il1b*. The effect of 2-DG on p-TEFb recruitment to *IL1B* further emphasizes intriguing connections between cell metabolism and transcription regulation of an important pro-inflammatory gene (Figure 8C). Our data provide evidence that *IL1B* and *TNF* promoters maintain paused Pol II for up to 25 h after their initial burst of transient transcription. Secondary stimulation re-recruits P-TEFb to *IL1B*, resulting in resumption and maintenance of transcription elongation in a manner more similar to classic IE genes. This contrasts tolerized *TNF*, in which secondary recruitment of P-TEFb and S2P modification of Pol II are absent, repressing the gene for extended time periods following vigorous transient transcription. Additional LPS stimulus can resume the transcription of the paused complexes on *IL1B* by means of gene specific P-TEFb recruitment, enabling resistance to endotoxin tolerance. Since nucleosome positioning controls promoter accessibility [64], we mapped promoter nucleosome distribution, as well as LPS-dependent temporal depletion and deposition at the end of the transient phase of *TNF* and *IL1B* transcription. Our data reveal cell type specific NDR upstream of the highly phased +1 nucleosome on both genes. Untransfected HEK293, which do not transcribe *IL1B*, revealed a highly phased −1 nucleosome within the NDR in the vicinity of the TSS. Transfection of Spi1 with TRAF6 and IRF8 (acting as an LPS surrogate) induces displacement of this −1 nucleosome. We hypothesize that this displaceable nucleosome serves as a control check-point mediating cell type and stimulus-dependent access to the transcription machinery at the *IL1B* promoter. Inhibition of transcription factor activation/recruitment to gene promoters in THP-1 cells similarly abolished LPS induced nucleosome clearance in a gene specific manner. While the inhibition of NF-κB had a pronounced effect on both *IL1B* and *TNF,* C/EBPβ inhibition only affected nucleosomes on the *IL1B* promoter. Our data provide a functional link between transcription factor activation and nucleosome clearance from these LPS-induced IE promoters.

Spatial-temporal analysis of chromatin modifications throughout the gene revealed monocyte-specific/stimulation-independent absence of inhibitory H3K27me3 throughout the entire length of both genes, contrasting the situation in cell types that do not transcribe these genes (Figure 3B). The H3K4me3 promoter mark present at the +1 nucleosome on *TNF* in unstimulated cells did not significantly increase 1 h post-stimulation. However, significant enrichment was observed at +1 and extending throughout the transcribed gene body during shutdown at 5 h. We argue that high levels of transcribing polymerases impede nucleosome deposition and modification throughout the transcribed region. At the end of transient transcription, nucleosomes were observed to be re-deposited to their original positions and became subject to histone modifiers. Our data reveal that the cell type-restricted expression of *IL1B* is due to the presence of the monocyte-specific differentiation factor Spi1, which binds constitutively to the *IL1B* promoter and enhancer in resting THP-1, poising the gene for induction. This binding is necessary, but insufficient, for LPS-mediated *IL1B* induction in THP-1 cells, as well as in HEK293 cells for which Spi1 in the absence of surrogate stimulation does not cause strong nucleosome clearance. We speculate that stimulation-dependent binding of NF-κB and C/EBPβ to the DNA loop-dependent proximity of constitutively bound Spi1, facilitates induction of *IL1B via* nucleosome remodeling. This is especially true for the −1 nucleosome, which appears to occlude TSS-proximal binding of TBP. This contrasts with *TNF*, in which the −1 nucleosome resides further upstream, permitting TBP access. The mechanism by which this occurs could depend upon the observed stimulation-dependent recruitment of p300 histone acetyltransferase (Figure 6A) and the SNF2β/BRG1 SWI/SNF chromatin remodeling enzyme (Figure S5J) by activated transcription factors. Both NF-κB [65], [66] and C/EBPβ [67], [68], as well as HIF-1 [69], [70] have been reported to directly recruit both SWI/SNF remodelers and p300 histone acetyltransferases. This would enable the nucleosome clearance required for Spi1-assisted recruitment of TBP to TATA box DNA. Regardless, as suggested by ectopic expression in HEK293 cells (Figure 4E), nucleosome remodeling depends upon the integrity of the Spi1 N-terminal domain in concert with the activation of key transcription factors, and appears to be necessary for TBP recruitment. These cooperative associations facilitate the subsequent assembly of the paused Pol II complex and regulate its release by P-TEFb in order to transition into productive elongation. The presence of highly dynamic Pol II further enhances the open promoter by competing with nucleosome re-deposition [71].

In summary, *IL1B* and *TNF* differ in the initial promoter state for unstimulated cells, with Spi1 and TBP possibly playing central roles for *IL1B*. Strikingly, during maximal initial expression (1 h) the chromatin architecture of the two genes looks quite similar. However, at 5 h distinct new architectures are established, resulting in *TNF* tolerance and establishing paused Pol II competent for re-stimulation on *IL1B* (Figure 8B). Importantly, we observe that these two NF-κB-dependent genes reveal numerous distinctions that may be reflective of known differences that exist for the cell source and function of their gene products. IL-1β protein expression is known to be more restricted to monocytes than is TNFα [37], likely dependent upon the requirement for Spi1 and its role in *de novo* recruitment of TBP. *IL1B* is also dependent upon LPS-activated C/EBPβ, which cooperates with NF-κB and Spi1 to induce transcription, likely in the context of a specific chromatin architecture involving the interaction between the promoter and a distal far-upstream enhancer.

It remains unclear, other than the requirement for Spi1 on *IL1B*, which factors are truly relevant for the priming of LPS responsive enhancers. The induction of *TNF* in cells which do not express Spi-1, argues against a universal role for Spi1 in LPS priming. LPS signal transduction involves Toll-IL-1 Receptor (TIR) signaling to activate pan-specific transcription factors, such as NF-κB and c-Jun [72] *via* IκB and MAP kinases, shared by various receptors found on a wide variety of cells. TLR4, the primary LPS receptor, is functional on a variety of non-myeloid cells, including basophils, keratinocytes, and epithelial cells [73]. NF-κB is also abundant in numerous cell types and plays a critical activation role in both *TNF* and *IL1B* gene induction. However, *IL1B* induction in monocytes also requires C/EBPβ, a protein more widely expressed than Spi1, but highly expressed in monocytic cells. Consequently, LPS signaling is not restricted to the myeloid/macrophage lineage, arguing that LPS-specific genomic programming may only require the appropriate receptor/signaling pathway and a receptive target gene. Therefore, priming of the gene may only be dependent upon its ability to present an open NDR promoter for Pol II recruitment. For *IL1B* in monocytes exposure may be primarily the binding of Spi1. In the case of *TNF*, promoter exposure may be accomplished either by monocyte or non-monocyte transcription factors, depending upon the cell-type.

The distinct functions of TNFα and IL-1β proteins are supported by the recent advent of specific therapeutic blockers, which reveal that there are diseases in which one or the other results in asymmetric efficacy, and occasionally asymmetric contraindication [74], [75], [76]. This is somewhat surprising, since both proteins activate similar signaling pathways in target cells. Consequently, it is reasonable that such functional differences might result from the differential gene regulation for two similar, but non-identical, immune effectors.

## Materials and Methods

### Cell Culture

THP-1 and Hut102 (ATCC) were cultured in RPMI. RAW264.7 (ATCC) were cultured in DMEM. HEK293 and MG63 (ATCC) were cultured in EMEM. All media was from Corning-Medaitech Cellgro. Cultures were all supplemented with 10% heat-inactivated fetal bovine serum (FBS, Hyclone), 1% Penicillin/Streptomycin Solution (Cellgro 30-002-CI). THP-1 cultures also contained 0.05 mM 2-mercaptoethanol (21985-023, Invitrogen). Adult human elutriated monocytes (Advanced Biotechnologies) and were cultured in DMEM with 20% FBS (Fisher), 1% Penicillin/Streptomycin and 50 µg/ml Gentamicin (MP Biomedicals) for 7 days until macrophage monolayer was established. On day 7 and 8, 90% of the old media was replaced with 10 ml of fresh media to remove all non-adherent cells. LPS stimulation was conducted on day 9 of cell culture. Murine bone marrow-derived macrophages (BMDM) from C57BL/6 mice (Harlan Laboratories, UK) were differentiated for 7 d in M-CSF (20% v/v) and L929 mouse fibroblast supernatant prior to experimental treatments. The BMDM were stimulated with 100 ng/ml LPS plus or minus 2–DG (1 mM) pretreatment for 3 h. All experiments involving mice were carried out with prior ethical approval from the Trinity College Dublin Animal Research Ethics Committee.

### Reagents and Treatment Conditions

In all experiments, monocytes were stimulated with 1 µg/ml of *E. coli* 055:B5 lipopolysaccharide (LPS) (Sigma) for indicated time periods. In the case of re-stimulation experiments, cells were initially stimulated with 1 µg/ml of LPS and then re-stimulated with additional 1 µg/ml of LPS without washing the media. All inhibitors used in the study were applied 1 h prior to LPS treatments in following concentrations; 1 µM/ml MG (Calbiochem), 10 µM/ml U0126 (Promega), 50 µM/ml 5,6-Dichlorobenzimidazole 1-β-D-ribofuranoside (DRB) (Sigma) 10 µM/ml BMS-345541 I KK Inhibitor III (Calbiochem) and 25 µM LY294002 (Calbiochem).

### Chromatin Immuno-precipitation (ChIP)

ChIP was performed using a modification of the Millipore/Upstate protocol (MCPROTO407). Fold enrichment was calculated based on Ct as 2^(ΔCt)^, where ΔCt = (Ct _Input_ – Ct _IP_). Final enrichment values were adjusted by subtraction of the nonspecific IgG antibody binding. Condensed data profiles are presented for many figures. Detailed statistical results for all such profiles appear in as Supporting Information, as reference in text. A total of 1×10^7^ cells were fixed in 1% formaldehyde (Fisher) for 10 min at room temperature. Cross-linking was inhibited by addition of glycine to a final concentration 0.125 M. Samples were sonicated (to generate DNA fragments of 250 base pairs (bp) average length) on ice using a Fisher Scientific Sonic Model 100 Dismembrator, as follows: 15×25 strokes at 100% power followed by 3×25 stokes at 50% power and centrifuged at 12000 RPM for 10 min. Chromatin from 5×10^6^ cells was diluted 7-fold in ChIP Dilution Buffer (0.01% SDS, 1.1% Triton X-100, 1.2 mM EDTA, 16.7 mM Tris-HCl, pH8.1, 167 mM NaCl), pre-cleared with protein Agarose/Salmon Sperm DNA beads (Protein G Agarose, 16–201 Millipore, Protein A Agarose 16–157 Millipore; IgM A4540 Sigma-Aldrich) for 30 min at 4°C, and centrifuged at 10,000 RPM for 2 min. Chromatin supernatants were incubated at 4°C overnight with respective antibodies (Table S2 in File S1). Aliquots for INPUT and non-specific IgG control samples were included with each experiment. Primer pairs against various regions of genes were designed using the PrimerQuest software available at the Integrated DNA technologies website (Tables S3–S8 in File S1). The size of the PCR products ranges between 67 and 192 bp overall (67 and 94 bp in the immediate vicinity of the *IL1B* promoter). Twenty microliter qPCR reactions containing 2x Maxima SYBR Green/ROX qPCR Master Mix (K0223, Fermentas), 250 nM of primers and 3 µl of precipitated DNA were set up in Fast 96-Well Reaction Plates (Applied Biosystems). qPCR reactions were carried out in a StepOnePlus Applied Biosystems Real Time Instrument. High-density overlapping qPCR amplicons, generated using closely-spaced primers (Tables S3, S5 in File S1) and fine fragmentation of chromatin [77], provided sufficient resolution for ChIP analysis to permit reproducible relative discrimination of peak maxima that exceed that which would be derived from the analysis of a single isolated PCR primer. However, it should be emphasized that although the x-axis positions are not absolute, specific locations, such as the binding sites for Spi1, TBP, and NF-κB are precisely known from previous studies [6], [9], [10]. These precisely located binding sites, which are associated with highly characterized nucleotide sequences serve as positional reference points. Such reference points are critical with respect to the results presented in Figure 3A, where the relative distribution of the peaks with respect to each other and the known reference points are not only reproducible, but also correspond to the distributions commonly observed in the vicinity of transcription initiation for a majority of metazoan genes. A minimum of two and a maximum of four completely independent experimental replicates were executed. However, the data presented are single experiments representative of the results.

### RNA Expression Analyses

1×10^6^ cells were plated into 6-well plates (Falcon). Following stimulation, cell pellets were resuspended in 500 µl of TRIzol reagent (Invitrogen). RNA was converted to cDNA using GoScript Reverse Transcription System (Promega A5001). Specific primers (Table S7 in File S1) were used to quantify gene expression *via* qPCR, as described above for ChIP. Relative mRNA levels were calculated using ΔΔCt method using b β-2-microtubulin and *18srRNA* as endogenous controls, and presented as the ratio in resting *vs*. LPS-treated cells. In certain experiments RNA was directly subjected to an RT-PCR utilizing the Access RT-PCR system (Promega). After addition of 170 µl of Chloroform (C606-1, Fisher) samples were vortexed, incubated at room temperature for 15 min, and centrifuged for 15 min at 13000 RPM in 4°C. Aqueous layer was removed, combined with equal volume of Isopropanol (BP2632-4, Fisher), 1 µl of Glycogen (9510, Ambion) and centrifuged for 10 min 13000 RPM at 4°C. Sample pellets were washed with 500 µl of 75% Ethanol (Pharmaco-AAPER) and centrifuged for 10 min in room temperature at 14000. Air-dried pellets were resuspended in 30 µl of RNAse free water and subjected to DNAse treatments using Turbo DNA-*free* reagents (AM1907, Ambion) according to the manufacturer instructions in order to eliminate genomic DNA contamination.

### *In Vitro* Protein Interaction Assays

GST-Spi1 fusion proteins were generated as previously reported [78].

### Transfection Constructs

Luciferase reporter XT-Luc *IL1B*, wild type IRF8 and mutant IRF8Y211F were as described [42]. Expression plasmids for wild-type C/EBPβ and the truncated C/EBPβΔSPL, were constructed and characterized as reported [8]. Expression plasmids expressing wild-type Spi1 and the dnSpi1 deletion mutant were constructed as described [9], [79]. The MHCκB-Luc reporter is as described [80], [81].

### Transient Transfection

HEK293 cells were seeded into 24 well plates to 60–70% confluency. Reporter and expression plasmids were transfected with FUGENE 6 Transfection Reagent (Roche 11814443001) at 3 µl of reagent per µg of DNA, according to the manufacturer’s instructions. Individual expression vectors were transfected as follows: 0.05 µg of Spi1 and 0.1 ug of TRAF6, IRF8, C/EBPβ and NF-κB into 24 well plates containing 500 µl of media. Total amount of transfected DNA was maintained constant for each experiment by addition of empty vector. Endogenous *IL1B* studies were conducted in 6 well culture plates with the amount of transfected material adjusted 3 fold.

### Luciferase Assays

At 24 h after transfection, cells were lysed with 60 µl of 1X cell lysis buffer in each well (24 well plate) and shaken for 20 min at RT. 20 µl of supernatant from each well was used for luciferase assay using Luciferase Assay System (Promega E1501) and analyzed by Veritas Microplate Luminometer and Software.

### Chromatin Conformation Capture (3C)

3C was performed using a modification of the protocol described in [82]. A total of 1.5×10^6^ cells were fixed in 2% formaldehyde (Fisher) for 10 min at room temperature. Cross-linking was inhibited by addition of glycine to a final concentration 0.125 M. Cell pellets were collected into 15 ml Falcon tubes and washed twice with ice cold PBS and resuspended in Lysis Buffer (10 mM Tris-HCl pH 8.0, 10 mM NaCl, 0.2% NP-40, supplemented with 1 mM PMSF and protease inhibitor cocktail (Sigma) in 1∶500 dilution) on ice for 90 min. Samples were centrifuged at 1800 rpm for 5 min, resuspended in 900 µl of 1.2×NEB4 (diluted with 0.3% SDS), and transferred into 1.5 ml Eppendorf tubes. Nuclear lysates were incubated for 1 hr at 37°C with moderate vortexing. 180 µl of Triton X-100 (final concentration of 1.8%) was added and samples were incubated for additional 1 hr at 37°C. Portion of chromatin (1 ug) was removed and treated overnight with MfeI (40 Unit) at 37°C. Lysates were treated with 1.6% SDS and incubated 60°C for 20 min. 47.5 µl of Lysates were used for ligation reaction (40 µl 10% Triton X-100, 40 µl ligase buffer (10x), 270 µl H_2_O, 2.5 µl T4 DNA ligase) that was carried out for 16 hours at 16°C. Next, 100 ug/ml of proteinase K was added to samples that were subsequently incubated overnight at 65°C. Next day, samples were treated with RNase A (0.5 ug/ml) for 30 min at 37°C and DNA was extracted. PCR products were amplified using GoTaq PCR Core System I (M7660, Promega) and analyzed by 2% agarose gel electrophoresis. Resolved fragments were then eluted and subjected to DNA sequencing in order to verify the identity of the ligation products.

### Site Directed Mutagenesis

XT-Luc binding site mutation reporter constructs: C/EBPβ I region binding site (XT-I c/g-Luc); NF-ΚB site at position −300 (XT-300κB-Luc); and the double I(c/g)/−300 κB were generated using QuickChange XL Site-Directed Mutagenesis Kit (Stratagene 200516) using appropriately mutated primer sequences.

### Western Blotting

Western blots were performed by standard protocols. Protein concentrations were determined using Pierce BCA Protein Assay Kit (Thermoscientific 23227).

## Supporting Information

Figure S1 **IL-1 and TNFα mRNA Expression Measured by QPCR in Various Cell Types.** (A) LPS-treated human THP-1 monocytes (Low resolution 0–27 h kinetics). (B) LPS-treated *ex vivo*-differentiated mouse peripheral blood monocytes. (C) LPS-treated mouse RAW264.7 monocytes. (D) Unstimulated human THP-1 monocytes evaluated for splicing using informative primers. (E) LPS-treated human THP-1 monocytes (High resolution 0–3 h kinetics).
(TIF)

Figure S2 **Pol II and TBP ChIP Comparing Human and Mouse Genes from LPS-treated Cells.** (A) Comparison of Pol II and TBP occupancy kinetics on *Il1* and *Tnf* genes for LPS-treated mouse RAW264.7 monocytes. (B) Representative bar graphs used to generate plots for Pol II ChIP in Figures 1C and S2A. (C) Pol II occupancy kinetics on *JUNB* and *HIST1H4K* genes for LPS-treated human THP-1 monocytes.
(TIF)

Figure S3 **ChIP Comparing Occupancy of Various General Transcription Factors and Modifications from LPS-treated Cells.** (A) LPS-treated RAW264.7 monocytes (Averaged profiles). (B) LPS-treated *ex vivo*-differentiated mouse BMDM (Averaged profiles derived from data shown in Figure 7B).
(TIF)

Figure S4 **ChIP used for Comparative Nucleosome Analysis of** ***IL1B*** **and** ***TNF*** **in Various Cells.** Summary profiles comparing nucleosome modifications for LPS-treated human THP-1 cells with untreated HEK293 pre-neuronal cells, Hut102 cutaneous T lymphocytes and MG63 osteoblastic cells.
(TIF)

Figure S5 **Transcription Factor-mediated Looping Between the** ***IL1B*** **Distal Enhancer and Promoter May Depend Upon the Binding of C/EBPβ.** (A) *IL1B* mRNA expression in HEK293 cells transfected with various expression vectors. (B) *IL1B* and *TNF* mRNA expression in HEK293 cells transfected with Spi1 expression vector. (C) *IL1B* and *TNF* mRNA expression kinetics in THP-1 cells treated with MG132 NF-κB/proteosome inhibitor. (D) Effect of IKKβ inhibitor on *IL1B* and *TNF* mRNA expression in THP-1 monocytes. (E) Effect of various inhibitors on *IL1B* and *TNF* mRNA expression in THP-1 and RAW264.7 monocytes. (F) IL1BXT-Luc reporter activity for ectopic expression of indicated factors transfected into HEK293. (G) IL1BXT-Luc and MHCκB reporter activity in RAW264.7 transfected with IκBα super repressor (IκBαSR). (H) Controls for Spi1 ectopic expression in transfected HEK293. (I) Glutathione S-transferase pull-downs demonstrate *in vitro* protein-protein interaction between the DNA binding domain of Spi1 and various transcription factors, including NF-κBp65.
(TIF)

File S1 Table S1. Relative levels of −1 and +1 nucleosome marks in relationship to amount of H3 on *IL1B* in THP1 cells. Table S2. Antibodies used for ChIP and Western blots. Table S3. Human *TNF* primer sequences. Table S4. Human *JUNB* and *HIST1H4K* primer sequences. Table S5. Human *IL1B* primer sequences. Table S6. Murine *Il1b* and *Tnf* ChIP primer sequences. Table S7. mRNA analysis and qPCR primer sequences. Table S8. Human *IL1B* 3C primer sequences.
(PDF)
