# Supplementary figures and images for: Distinct Mechanisms for Induction and Tolerance Regulate the Immediate Early Genes Encoding Interleukin 1β and Tumor Necrosis Factor α
Source: PLoS One. 2013 Aug 1;8(8):e70622. doi: 10.1371/journal.pone.0070622 (PMC3731334; doi:10.1371/journal.pone.0070622)

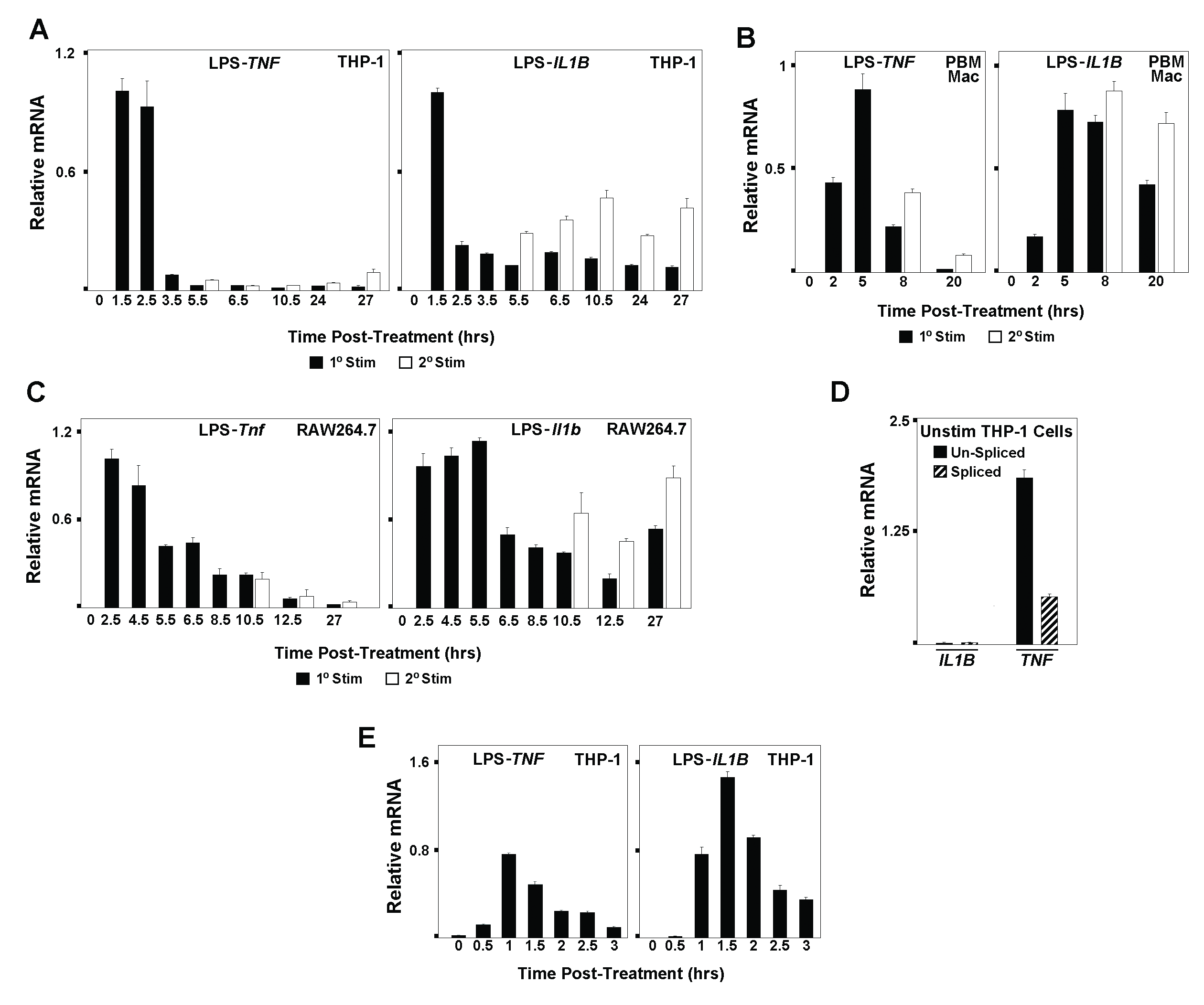

Supplement: Figure S1 — IL-1 and TNFα mRNA Expression Measured by QPCR in Various Cell Types. (A) LPS-treated human THP-1 monocytes (Low resolution 0–27 h kinetics). (B) LPS-treated ex vivo-differentiated mouse peripheral blood monocytes. (C) LPS-treated mouse RAW264.7 monocytes. (D) Unstimulated human THP-1 monocytes evaluated for splicing using informative primers. (E) LPS-treated human THP-1 monocytes (High resolution 0–3 h kinetics). (TIF) [file pone.0070622.s001.tif]

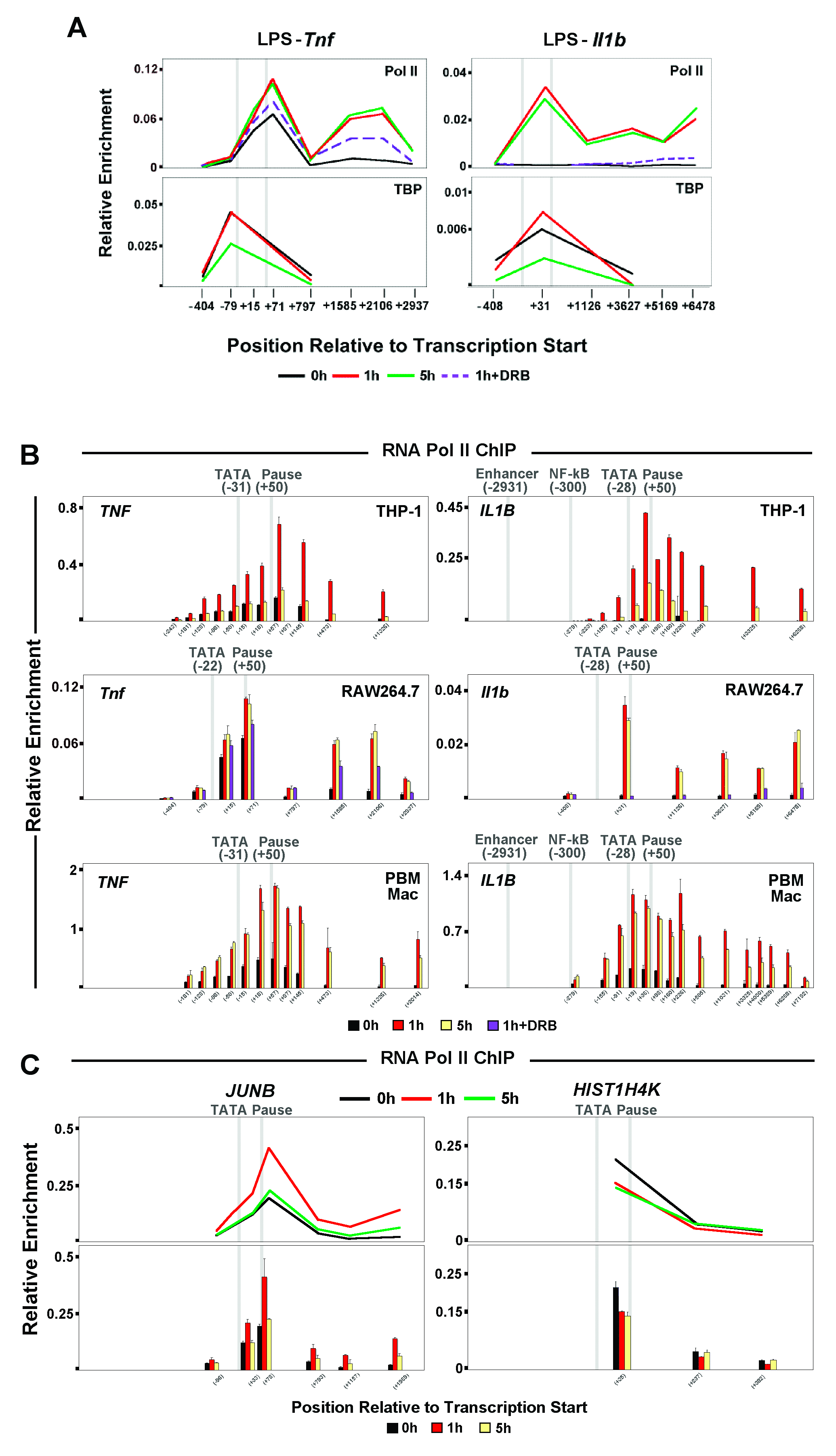

Supplement: Figure S2 — Pol II and TBP ChIP Comparing Human and Mouse Genes from LPS-treated Cells. (A) Comparison of Pol II and TBP occupancy kinetics on Il1 and Tnf genes for LPS-treated mouse RAW264.7 monocytes. (B) Representative bar graphs used to generate plots for Pol II ChIP in Figures 1C and S2A. (C) Pol II occupancy kinetics on JUNB and HIST1H4K genes for LPS-treated human THP-1 monocytes. (TIF) [file pone.0070622.s002.tif]

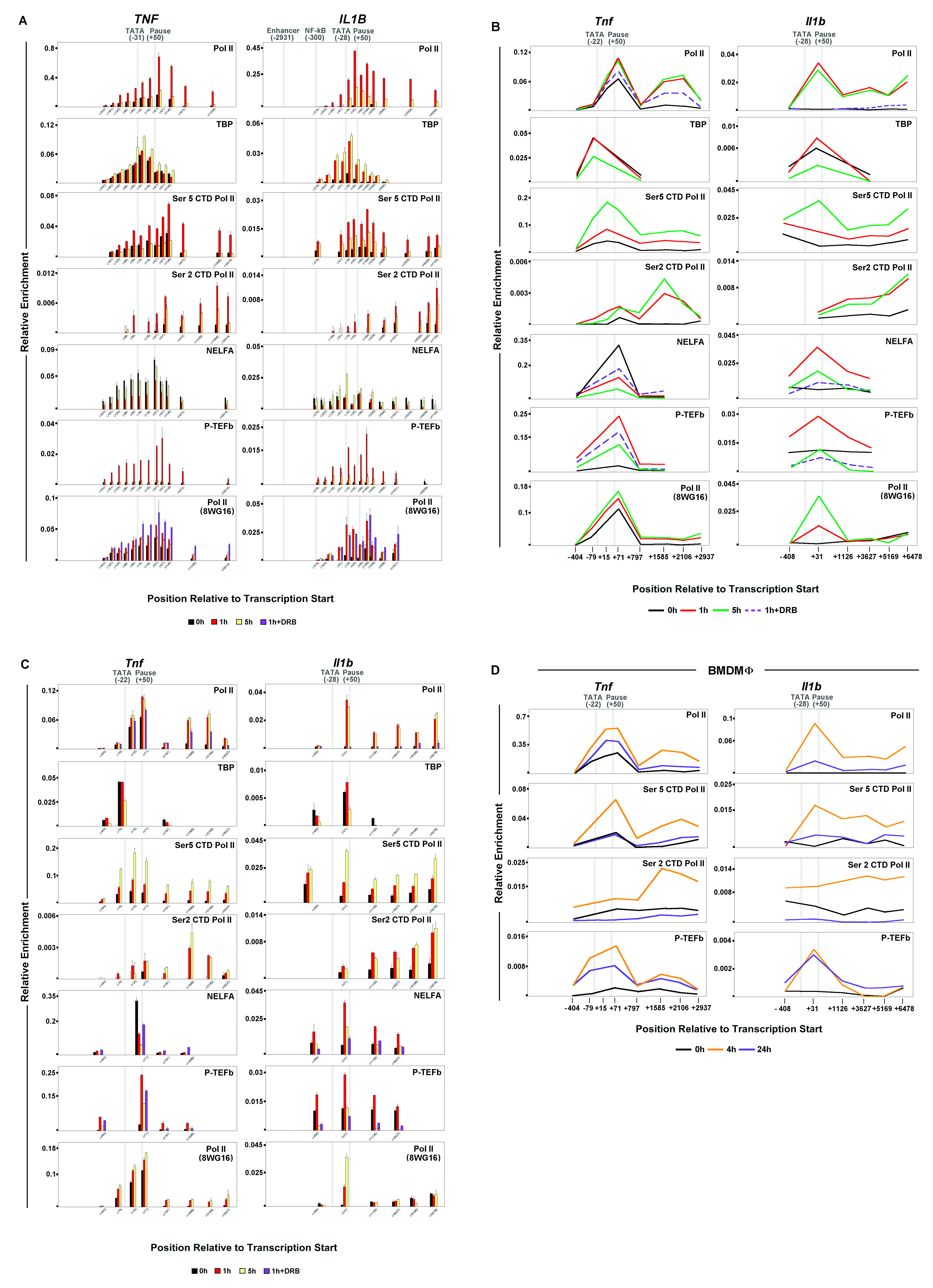

Supplement: Figure S3 — ChIP Comparing Occupancy of Various General Transcription Factors and Modifications from LPS-treated Cells. (A) LPS-treated RAW264.7 monocytes (Averaged profiles). (B) LPS-treated ex vivo-differentiated mouse BMDM (Averaged profiles derived from data shown in Figure 7B). (TIF) [file pone.0070622.s003.tif]

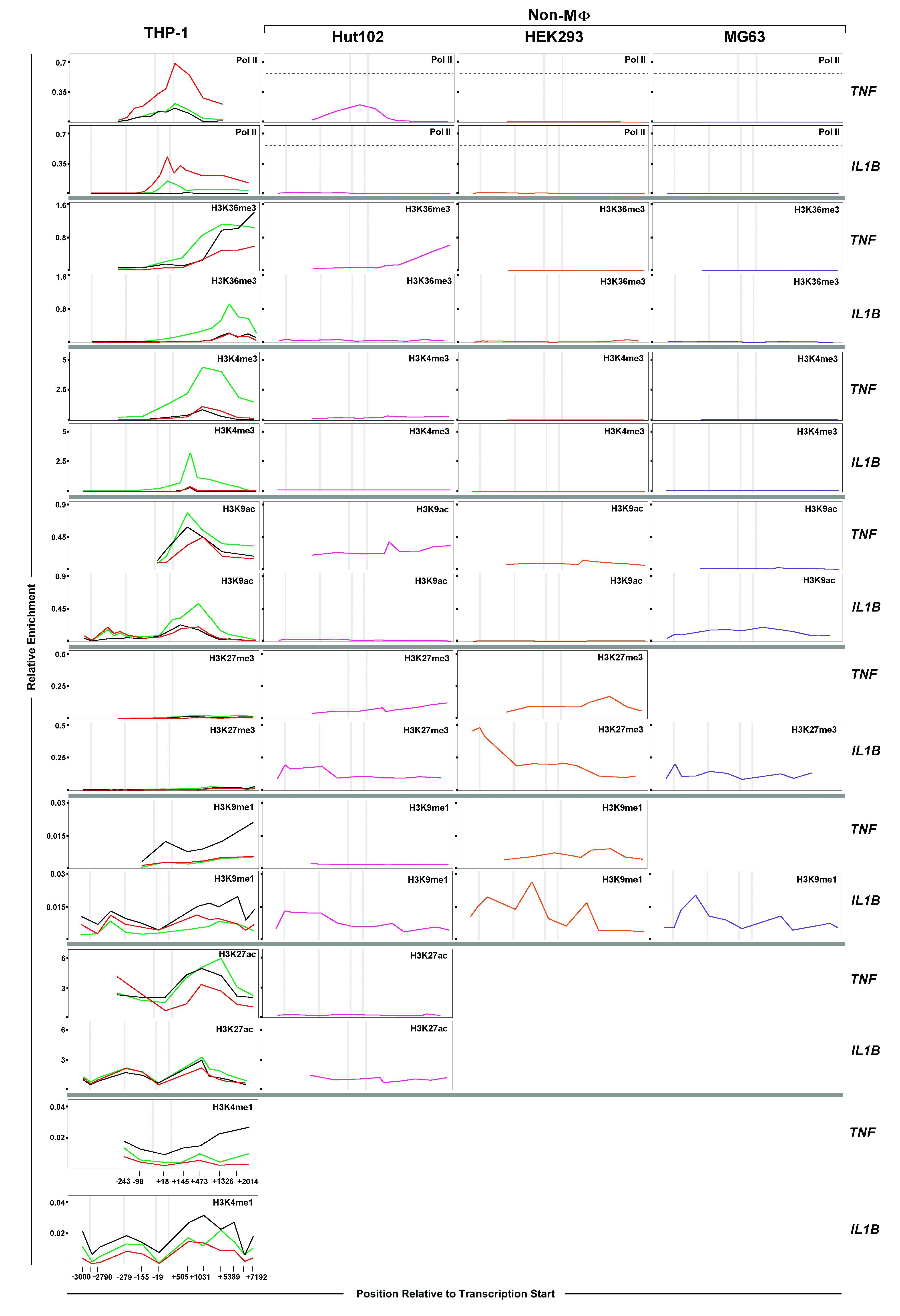

Supplement: Figure S4 — ChIP used for Comparative Nucleosome Analysis of IL1B and TNF in Various Cells. Summary profiles comparing nucleosome modifications for LPS-treated human THP-1 cells with untreated HEK293 pre-neuronal cells, Hut102 cutaneous T lymphocytes and MG63 osteoblastic cells. (TIF) [file pone.0070622.s004.tif]

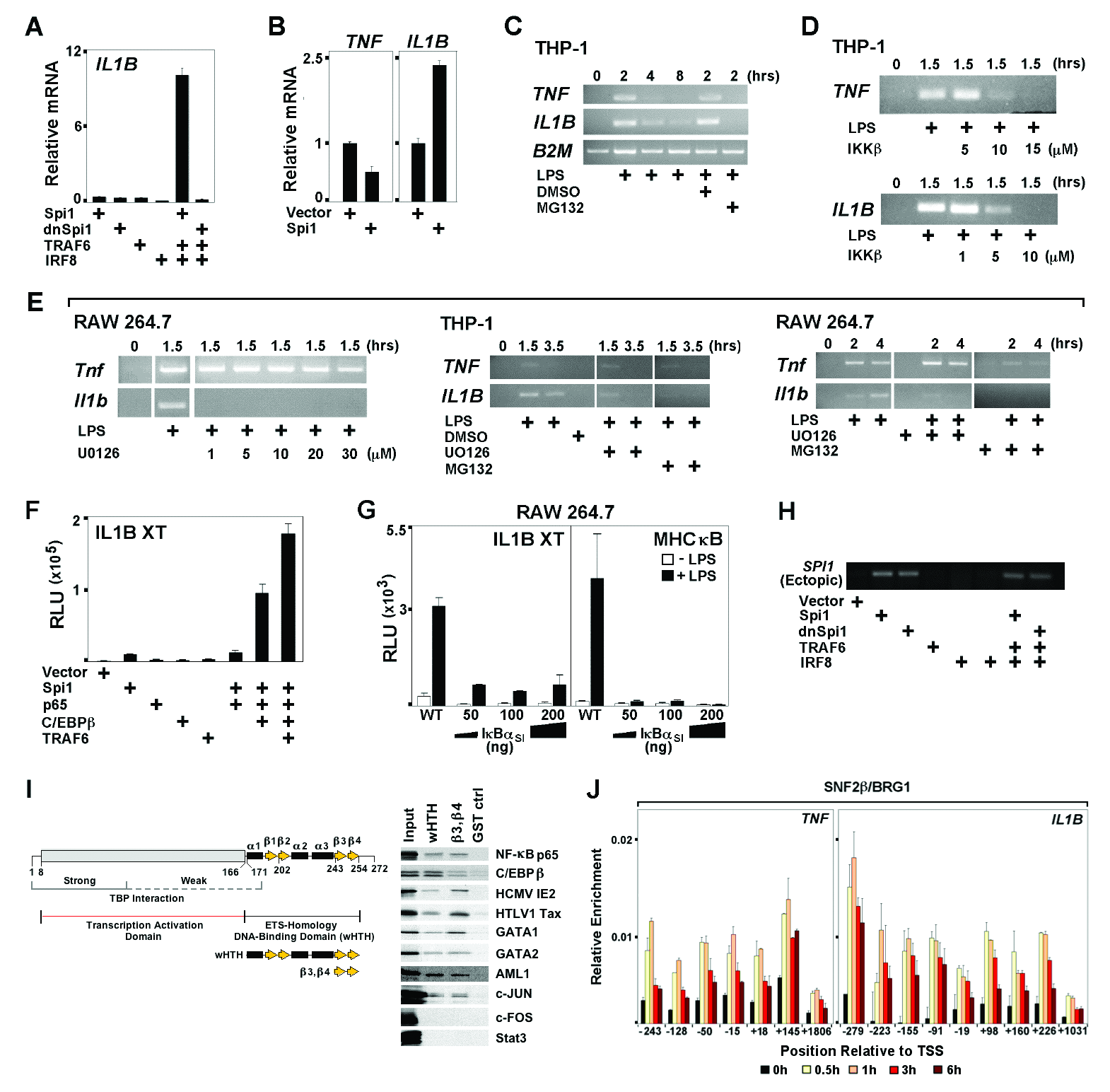

Supplement: Figure S5 — Transcription Factor-mediated Looping Between the IL1B Distal Enhancer and Promoter May Depend Upon the Binding of C/EBPβ. (A) IL1B mRNA expression in HEK293 cells transfected with various expression vectors. (B) IL1B and TNF mRNA expression in HEK293 cells transfected with Spi1 expression vector. (C) IL1B and TNF mRNA expression kinetics in THP-1 cells treated with MG132 NF-κB/proteosome inhibitor. (D) Effect of IKKβ inhibitor on IL1B and TNF mRNA expression in THP-1 monocytes. (E) Effect of various inhibitors on IL1B and TNF mRNA expression in THP-1 and RAW264.7 monocytes. (F) IL1BXT-Luc reporter activity for ectopic expression of indicated factors transfected into HEK293. (G) IL1BXT-Luc and MHCκB reporter activity in RAW264.7 transfected with IκBα super repressor (IκBαSR). (H) Controls for Spi1 ectopic expression in transfected HEK293. (I) Glutathione S-transferase pull-downs demonstrate in vitro protein-protein interaction between the DNA binding domain of Spi1 and various transcription factors, including NF-κBp65. (TIF) [file pone.0070622.s005.tif]
