# Supplementary material for: Distinct Mechanisms for Induction and Tolerance Regulate the Immediate Early Genes Encoding Interleukin 1β and Tumor Necrosis Factor α
Source: PLoS One. 2013 Aug 1;8(8):e70622. doi: 10.1371/journal.pone.0070622 (PMC3731334; doi:10.1371/journal.pone.0070622)
Supplement: File S1 — Table S1. Relative levels of −1 and +1 nucleosome marks in relationship to amount of H3 on IL1B in THP1 cells. Table S2. Antibodies used for ChIP and Western blots. Table S3. Human TNF primer sequences. Table S4. Human JUNB and HIST1H4K primer sequences. Table S5. Human IL1B primer sequences. Table S6. Murine Il1b and Tnf ChIP primer sequences. Table S7. mRNA analysis and qPCR primer sequences. Table S8. Human IL1B 3C primer sequences. (PDF) [file pone.0070622.s006.pdf]

Table S1. Relative Levels of -1 and +1 Nucleosome Marks in Relationship to Amount of H3 on *IL1B* in THP1 Cells

Nucleosome	Mark	*TNF*			*IL1B*		
		0 h	1 h	5h	0 h	1 h	5 h
+1 Nucleosome	H3K4me3	0.19	0.56	0.75	0.02	0.03	0.24
+1 Nucleosome	H3K9ac	0.83	1.61	0.98	0.28	0.31	0.65
+1 Nucleosome	H3K27me3	0.04	0.17	0.08	0.02	0.03	0.06
+1 Nucleosome	H3K36me3	0.21	0.56	0.56	0.02	0.03	0.17
-1 Nucleosome	H3K4me3	0.05	0.13	0.62	0.06	0.09	0.50
-1 Nucleosome	H3K9ac	nd	nd	nd	0.06	0.64	1.33
-1 Nucleosome	H3K27me3	0.05	0.13	0.08	0.06	0.09	0.17
-1 Nucleosome	H3K36me3	0.20	0.25	0.31	0.06	0.09	0.42

Table S2. Antibodies Used For ChIP and Western Blots

EPITOPE	SOURCE	CATALOG NUMBER	AGAROSE IP
RNA Pol II (N-20)	Santa Cruz Biotechnology, Inc.	sc-899	Protein A
RNA Pol II CTD Ser5	Babco-Covance	MMS-134R	Protein M
RNA Pol II CTD Ser2	Babco-Covance	MMS-129R	Protein M
RNA Pol II 8WG16	Babco-Covance	MMS-126R	Protein M
Cdk9 (H-169)	Santa Cruz Biotechnology, Inc.	sc-8338	Protein A
NELF-A (A-20)	Santa Cruz Biotechnology, Inc.	sc-23599	Protein G
PU.1 (H-135)	Santa Cruz Biotechnology, Inc.	sc-22805	Protein A
PU.1/Spi-1 (T-21)	Santa Cruz Biotechnology, Inc.	sc-352	Protein A
TFIID (TBP) (SI-1)	Santa Cruz Biotechnology, Inc.	sc-273	Protein G
TBP	Abcam, Inc.	ab28175	Protein A
C/EBPβ (47A1)	Santa Cruz Biotechnology, Inc.	sc-56637	Protein G
H3 (tri methyl K36)	Abcam, Inc.	ab9050	Protein A
H3 (tri methyl K27)	Active Motif	39156	Protein G
H3 (tri acetyl K27)	Abcam, Inc.	ab4729	Protein A
H3 (mono methyl K9)	Millipore	07-450	Protein A
H3 (acetyl K9)	Upstate	07-352	Protein A
H3 (mono methyl K4)	Active Motif	39635	Protein G
H3 (tri methyl K4)	Active Motif	39915	Protein A
Histone H3 (FL-136)	Abcam, Inc.	ab1791	Protein A
BRD4	Abcam	ab84776	Protein A
P300 (N15)	Santa Cruz Biotechnology, Inc.	Sc-584	Protein A/G^{+}
NFκB p65	Abcam, Inc.	ab7970	Protein A
Anti-proIL-1β	R&D Systems	MAB6964	(Western Blot)

Table S3. Human *TNF* Primer Sequences

Position	Forward	Reverse
-243	CACAAATCAGTCAGTGGCCCAGAA	GGACACACAAGCATCAAGGATACC
-181	GGTATCCTTGATGCTTGTGTGTCC	CTGCACCTTCTGTCTCGGTTTCTT
-128	GGAGAAGAAACCGAGACAGAAGGT	CTTCCTTGGTGGAGAAACCCATGA
-98	ACTACCGCTTCCTCCAGATGAG	GGGAAAGAATCATTCAACCAGCGG
-50	CCGCTGGTTGAATGATTCTT	TGTGCCAACAACTGCCTTTA
-15	CCCAGGGACATATAAAGGCAGTTG	TGTCCTTGCTGAGGGAGCGT
+18	TTGGCACACCCAGCCAGCAGA	GGTCTGTAGTTGCTTCTCTCCCTCTT
+57	CCAGCTAAGAGGGAGAGAAGCAACTA	ATGTGGCGTCTGAGGGTTGTTT
+97	AAACAACCCTCAGACGCCACAT	TATGTGAGAGGAAGAGAACCTGCC
+145	AGGCAGGTTCTCTTCCTCTCACAT	TGCTTTCAGTGCTCATGGTGTCCT
+214	TGGAAAGGACACCATGAGCACTGA	TGAGGAACAAGCACCGCCT
+293	AGGCGGTGCTTGTTCCTCA	TCCAAAGTGCAGCAGGCAGAAGA
+473	TGGGTGAAAGATGTGCGCTGATAG	TTGCCACATCTCTTTCTGCATCCC
+1326	ATGTGTCTTGGAACTTGGAGGGCT	TATCCCACTAAGGCCTGTGCTGTT
+1806	TCAACCTCCTCTCTGCCATCAAGA	ATAGATGGGCTCATACCAGGGCTT
+2014	AGGACGAACATCCAACCTTCCCAA	TTTGAGCCAGAAGAGGTTGAGGGT

Table S4. Human *JUNB* and *HIST1H4K* Primer Sequences

JUNB

Position	Forward	Reverse
-96	CCGCTGTTTACAAGGACACG	GGAAGTGCGCTCCGATTG
+33	GGCTGGGACCTTGAGAGC	GTGCGCAAAAGCCCTGTC
+78	TGACTAGCGCGGTATAAAGGCGT	CGATAGCTTTCCTGGCGTCGTTT
+793	GGACGATCTGCACAAGATGA	TGCTGAGGTTGGTGTAAACG
+1157	CATCAAAGTGGAGCGCAA	TTGAGCGTCTTCACCTTGTC
+1969	CCAGCTCAGTGCTGTTGGT	ATCCAACCCTGGAGATCTGG

HIST1H4K

+35	GACTCCTCTTGCTCGTCATGTCTG	CGCCTTTGCCAAGACCCT
+237	GGTGCTGAAGGTGTTCCTGG	CGCTTGGCGTGCTCTGTA
+382	GGTTGAGCGTCCCTTTCTATCAACA	TGGGCAAACAAGCATCACGG

Numbers indicate the midpoint of amplicons in relation to the TSS

Table S5. Human *IL1B* Primer Sequences

Position	Forward	Reverse
- 3000	CCAAGGAAGTGGAACAAGCAGAGA	CCAAGGAAGTGGAACAAGCAGAGA
- 2876	TCTCCTTGGGAAGACAGGATCTGA	ACCTGGACCCAAATCTGCTACCAT
- 2790	ACATGGCAGAACTGTGGAGACTGT	ATAGCACAGTGTGGTTGAAGCAGC
- 2329	TCCCTTCCTGTCACTGGCTTTGAT	TGCATGATCACAGCAGCCTCAAAC
- 1520	ATCAGAAGGCTGCTTGGAGAGCAA	TGCATTATCCAAGGGAGTGAGCCT
- 800	TAGGCAGAGCTCATCTGGCATTGA	ATTGCAGGAGCCTCTGAGGAGAAA
-279	TGTGTGTCTTCCACTTTGTCCCAC	CCTGACAATCGTTGTGCAGTTGATG
-223	TGTGGACATCAACTGCACAACG	TTCATGGAAGGGCAAGGAGTAGCA
-155	TTGCTACTCCTTGCCCTTCCATGA	GAGTATTGGTGGAAGCTTCTTAGGG
- 91	CCCTAAGAAGCTTCCACCAATACTC	GCAGAAGTAGGAGGCTGAGAAA
- 19	CCCTAAGAAGCTTCCACCAATACTC	GCAGAAGTAGGAGGCTGAGAAA
+ 36	AAACCTCTTCGAGGCACAAG	GAGCAATGAAGATTGGCTGA
+ 98	CAGCCAATCTTCATTGCTCA	GCATACACACAAAGAGGCAGAG
+ 160	CTCTGCCTCTTTGTGTGTATGC	GAGGGAAGGAGAGGGAGAGA
+ 223	TCTCCCTCTCCTTCCCTCTC	TTCCCAGAATATTTCCCGAGT
+ 271	GCCAGGTGTAATATAATGCTTATGACTCGG	GACACTAACCTTTAGGGTGTCAGC
+505	TGCACTGGATGCTGAGAGAAA	GGCTGCTTCAGACACCTGTG
+ 1031	AATCTGCCTTCTGGACTGTTCTGC	AAGAAACTGCAAACAGCCTGCCTC
+ 3325	AATCTCCGACCACCACTACAGCAA	AAGGGAAAGAAGGTGCTCAGGTCA
+ 4000	TCGACACATGGGATAACGAGGCTT	TGGAGGTGGAGAGCTTTCAGTTCA
+ 5389	ACTGCTGTGTCCCTAACCACAAGA	TTCAACACGCAGGACAGGTACAGA
+ 6268	TCGCTGCAGAGTGTAGATCCCAAA	TGCTTGAGAGGTGCTGATGTACCA
+ 7192	CCTCATTGCTGAGTGCTGCAAAGT	AGTGCTTCAGCTGATCCTGTTCCA

Numbers indicate the midpoint of amplicons in relation to the TSS

Table S6. Murine *Il1b* and *Tnf* ChIP Primer Sequences

Il1b

Position	Forward	Reverse
-408	TGATGGAGAGCACAGAAGCACCAT	ACGTAGATGCACACCCAGAAGTGT
+ 31	ACAAGTTGGACAACAAACCCTGC	TACTTGCACAAGGAAGCTTGGCTGGA
+ 1126	AGGGACTCCTACAGATGCAATGGT	TGCTCTGGTTGCTCTCTGTTGACT
+ 3627	AAATCCAATGTTCTTGCCCAGCCC	TGCAAGCACTGTGAAGTGAAGCAG
+ 5169	GCCTTGGGCCTCAAAGGAAAGAAT	TAAAGGCAGAGTCTTCGGTGAGCA
+ 6478	ATAGCCCGCACTGAGGTCTTTCAT	CTCATCAAAGCAATGTGCTGGTGC

Tnf

Position	Forward	Reverse
- 404	TATGCACCCAGCTTTCAGAAGCAC	AGGACCCTGAGAACTGAAACCCAT
- 79	ACTACCGCTTCCTCCACATGAGAT	AGACGGCCGCCTTTATAGCCCTT
+ 15	CTCTTCCCCAAGGGCTATAAAGG	CTTGCTTTTCTGGGAGCTATTTCC
+ 71	ACTAGCCAGGAGGGAGAACAGAAA	AGTGAGTGAAAGGGACAGAACCTG
+ 797	AAAGAAGCCGTGGGTTGGACAGAT	AGAACTGATGAGAGGGAGGCCATT
+ 1585	AGCCGATGGGTTGTACCTTGTCTA	TGAGATAGCAAATCGGCTGACGGT
+ 2106	TCTCATGCACCACCATCAAGGACT	ACCACTCTCCCTTTGCAGAACTCA
+ 2937	GTACTGCAGTTCTTTCCGGCTGTT	TCTGTCTGTTCAGAGGCAGGTTGA

Numbers indicate the midpoint of amplicons in relation to the TSS

Table S7. mRNA Analysis and qPCR Primer Sequences

Gene	Forward	Reverse
18srRNA	TAGAGGGACAAGTGGCGTTC	TCCTCGTTCATGGGGAATAA
hB2M	TGCTGTCTCCATGTTTGATGTATC	TCTCTGCTCCCCACCTCTAAGT
mB2M	GGCCTGTATGCTATCCAGAA	GAAAGACCAGTCCTTGCTGA
hIL1B	AATCTCCGACCACCACTACAGCAA	AAGGGAAAGAAGGTGCTCAGGTCA
Splice IL1B	TCTGTACCTGCTCTGCGTGTTGAA	GGGCAGACTCAAATTCCAGCTTGT
mIL1B	CCCAACTGGTACATCAGCAC	TCTGCTCATTCACGAAAAGG
hTNF	AAGCCCTGGTATGAGCCCATCTAT	ATGATCCCAAAGTAGACCTGCCCA
Splice TNF	AGTGACAAGCCTGTAGCCCATGTT	GTTATCTCTCAGCTCCACGCCATT
mTNF	CCCACTCTGACCCCTTTACT	TTTGAGTCCTTGATGGTGGT
hSPI1	CCAGCTCAGATGAGGAGGAG	GCTTGGACGAGAACTGGAAG
hC/EBPA	TGGCCAACTTCTACTACGAGG	AGAGGAAGTCGTGGTGCTGC
hC/EBPB	TACTACGAGGCGGACTGCTT	CGTAGTCGTCGGAGAAGAGG
p65	GGCGAGAGGAGCACAGATAC	CACTGTCACCTGGAAGCAGA
hIRF8	ACGAGGTTACGCTGTGCTTT	CCAGTCTGGAAGGAGCTGAC

Site directed mutagenesis primer sequences

IL1B enhancer I region XT-I (c/g)-Luc primer pairs:

I (c/g) F: 5'CTGTGGAGACTGTTAGGTCAGGGGGCATTGC3'
I (c/g) R: 5'GCAATGCCCCCTGACCTAACAGTCTCCACAG3'

NF-κB site (-300) mutation

XT-300 F:
5'AACATTCTTCTAACGTGTGAAAATACAGTATTTTAATGTGGACATC3'
XT-300 R:
5'GATGTCCACATTAAAATACTGTATTTTCACACGTTAGAAGAATGTT3'

I region / NF-κB site (-300) double mutant was engineered by using the XT-I(c/g)-Luc plasmid as a template and the XT-300mut(F)/(R) as primers.

Table S8. Human *IL1B* 3C Primer Sequences

Designation	Position	Sequence
1’	-12369 / -12349	GGGGCCTCCAAATCACTAAGC
2	-2772 / -2750	GCATTGCCCCATGGCTCCAAAAT
3’	- 482 / -504	TCTCTACCTTGGGTGCTGTTCTC
4	4605 / 4627	CCGCTGTAACGGGCAAAAGTTTC
